# Physiological and Metagenomic Analyses of Microbial Mats Involved in Self-Purification of Mine Waters Contaminated with Heavy Metals

**DOI:** 10.3389/fmicb.2016.01252

**Published:** 2016-08-10

**Authors:** Lukasz Drewniak, Pawel S. Krawczyk, Sebastian Mielnicki, Dorota Adamska, Adam Sobczak, Leszek Lipinski, Weronika Burec-Drewniak, Aleksandra Sklodowska

**Affiliations:** ^1^Laboratory of Environmental Pollution Analysis, Faculty of Biology, University of WarsawWarsaw, Poland; ^2^Laboratory of RNA Biology and Functional Genomics, Institute of Biochemistry and Biophysics, Polish Academy SciencesWarsaw, Poland; ^3^Polish Geological Institute-National Research InstituteWarsaw, Poland

**Keywords:** heavy metals, metagenomes, microbial mats, mine waters, self-purification, sorption

## Abstract

Two microbial mats found inside two old (gold and uranium) mines in Zloty Stok and Kowary located in SW Poland seem to form a natural barrier that traps heavy metals leaking from dewatering systems. We performed complex physiological and metagenomic analyses to determine which microorganisms are the main driving agents responsible for self-purification of the mine waters and identify metabolic processes responsible for the observed features. SEM and energy dispersive X-ray microanalysis showed accumulation of heavy metals on the mat surface, whereas, sorption experiments showed that neither microbial mats were completely saturated with heavy metals present in the mine waters, indicating that they have a large potential to absorb significant quantities of metal. The metagenomic analysis revealed that *Methylococcaceae* and *Methylophilaceae* families were the most abundant in both communities, moreover, it strongly suggest that backbones of both mats were formed by filamentous bacteria, such as Leptothrix, Thiothrix, and Beggiatoa. The Kowary bacterial community was enriched with the *Helicobacteraceae* family, whereas the Zloty Stok community consist mainly of *Sphingomonadaceae, Rhodobacteraceae*, and *Caulobacteraceae* families. Functional (culture-based) and metagenome (sequence-based) analyses showed that bacteria involved in immobilization of heavy metals, rather than those engaged in mobilization, were the main driving force within the analyzed communities. In turn, a comparison of functional genes revealed that the biofilm formation and heavy metal resistance (HMR) functions are more desirable in microorganisms engaged in water purification than the ability to utilize heavy metals in the respiratory process (oxidation-reduction). These findings provide insight on the activity of bacteria leading, from biofilm formation to self-purification, of mine waters contaminated with heavy metals.

## Introduction

Microbial mats are complex microbial communities, which are found in various ecological niches, e.g., on the surface of sediments, floating masses in fresh waters (Sanz-Montero and Rodriguez-Aranda, 2013; Hawes et al., 2014), marine waters, including deep ocean (Sudek et al., 2009; Kubo et al., 2012), hypersaline waters (Cantrell et al., 2013; Valdivieso-Ojeda et al., 2014), and geothermal springs (Coman et al., 2013; Klatt et al., 2013).

Among the different environments where microbial mats have been described, mine waters, especially those contaminated with heavy metals, are of special interest (Drewniak et al., 2012; Mendez-Garcia et al., 2014), However, such communities are usually described in the context of acid mine drainage (AMD), as microorganisms are often responsible for the formation of these effluents, which constitute a well-known environmental problem (Hogsden and Harding, 2012). Numerous studies apply metagenomic approach to investigate the phylogenetic and functional properties of microorganisms involved in AMD development (Chen et al., 2016; Huang et al., 2016) as well as metal-microbes interactions and biogeochemical pathways.

Furthermore, for some isolates and multi-species communities found in AMD, mechanisms of metals attenuation have been described (Lawrence et al., 1998; Morin et al., 2003; Gandy et al., 2007; Rowe et al., 2007; Sánchez-Andrea et al., 2012). On the other hand, there is a limited number of reports on microbial-driven attenuation of heavy metals in neutral mine waters (Podda et al., 2000).

Two old mines, the Zloty Stok goldmine (ZS), and Kowary uranium mine (KOW) located in South-Western Poland are interesting locations to study the metal-microbe interactions at the ecosystem level, as complex microbial mats communities were observed in the bottom sediments of both slightly acidic, and neutral mine waters with elevated concentrations of heavy metals.

Our preliminary (3-year) observation found that microbial mats from both mines are constantly renewing in their natural habitat, and most importantly, act as effective retaining filters of metals, and radionuclides that can immobilize most of the pollutants flowing through the mine water. The latter feature is of particular importance for environmental protection and bio-metallurgy, and is directly connected to the geochemical background of both mines.

ZS mine communities were already examined using low-throughput methods, including culturing of arsenic-transforming bacteria (Drewniak et al., 2008, 2010, 2015) or 16S rRNA amplification (Drewniak et al., 2012; Tomczyk-Zak et al., 2013), indicating their high potential for heavy metals utilization. However, they were never analyzed more deeply using novel techniques applying metagenomic sequencing. Our discovery of similar microbial mats in another abandoned mine (KOW) allowed us to compare both communities, and to determine whether they share a common mechanism for heavy metal attenuation (despite their geographical isolation).

Thus, the aim of this study was to investigate the sorption and transformation of heavy metals by those naturally-occurring microbial mats from slightly acidic or neutral waters and perform a comparative analysis of both microbial mat metagenomes in the context of heavy metal homeostasis in such extreme ecosystems. Physiological analyses of sorption and transformation capabilities were complemented with metagenomic description of microorganisms and the analysis of probable mechanisms involved in heavy metals attenuation. The results of this study, indicating shared properties of both communities, shed new light on the bioprocesses occurring in the mats and identify the microorganisms that play a pivotal role in self-purification of slightly acidic and neutral waters contaminated with heavy metals.

## Materials and methods

### Sampling sites description

The ZS and KOW mines are located in the Lower Silesia district of South-Western Poland (Supplementary Figure S1) in a region where mining and smelting activities have been ongoing for more than 800 years. ZS has been of the greatest gold mining centers in Europe since the thirteenth century. In addition, arsenic exploitation began there in the eighteenth century and lasted until 1962 when the mine was closed, leaving ~300 km of underground passages, mostly filled with water, partly, or totally inaccessible (Drewniak et al., 2010). Arsenic is currently the main contaminant of soils as well as surface and ground waters present in the post-mining region. The mining activity in the KOW area began in 1148 for the procurement of magnetite ore. Polymetallic minerals and uranium were subsequently detected and mined starting around 1910. Currently, the KOW mining remnants consist of several dumps and kilometers of underground galleries filled with water, similar to ZS.

Several adits (galleries) in both mines function as an efficient dewatering systems that discharge mine water directly into the Golden Stream at ZS and Jedlica River at KOW. However, water from other shafts infiltrate ground, and surface water in the vicinity of both mines, resulting in serious contamination with heavy metals of the surrounding area. Pollution disseminated in the flowing surface waters also poses a danger to drinking water sources. However, the microbial mats that inhabit bottom sediments of the adits form a natural barrier that traps heavy metals leaking from the dewatering system of the mines.

### Sample collection and chemical analysis

Microbial mat samples were collected from the rocks located on sediments of mine streams present in the following transportation corridors: Gertruda adit in the ZS gold mine and adit no. 19a in the KOW uranium mine. Microbial mat samples were collected with the mine water samples and transported to the laboratory at 4°C. Microbial mat samples were collected in three separate sampling runs and were analyzed independently.

For chemical (elemental) analysis samples of each microbial mat were thoroughly dried for 24 h at 60°C and pulverized in an agate mortar. Samples were subsequently digested with 5 mL 65% HNO_3_, and 1 mL 30% H_2_O_2_ per 0.1 g of dry mass in a closed system with heating in a microwave oven (Multiwave 3000, Anton Paar).

The concentrations of arsenic and uranium as well as other elements (Al, Cd, Co, Cr, Cu, Fe, Mo, Mn, Ni, Pb, V, and Zn) present in the microbial mats were analyzed by inductively coupled plasma optical emission spectrometry (ICP-OES; iCAP6500 DUO, Thermo Scientific). Quantitative analysis of Al, As, Ca, Cd, Co, Cr, Cu, Fe, Mn, Mo, Ni, Pb, V, U, and Zn from mine water samples was performed with inductively coupled plasma mass spectrometry (ICP-MS; ELAN DRC II, Perkin Elmer). Arsenic species were separated by HPLC (Waters Alliance) on an RP Waters IC-PakTM Anion HC column (150 by 4.6 mm; pore size, 10 μm) and quantified by Graphite Furnace Atomic Absorption Spectrometry (GFAAS; AA Solaar M6 Spectrometer, TJA Solutions). The reduction of Fe^3+^ ions was monitored by a test with 1,10-phenanthroline (Edwards et al., 1975), and the Fe^2+^ ion concentration was measured spectrophotometrically at 510 nm (Shimadzu UV-1800). A Nanocolor Sulfat 200 test (Macherey-Nagel) was used to determine the ${\text{SO}}_{4}^{2-}$ ion concentration.

### Metagenomic DNA isolation, sequencing, and analysis

Total DNA was extracted from microbial mat and enrichments culture samples as previously described by Drewniak et al. (2012). Approximately 50–60 μg of DNA was isolated from each 10 mL microbial mat sample and 100 mL of enrichments cultures.

Metagenomic DNA extracted from microbial mats was used for preparation of shotgun metagenomic libraries. The libraries were prepared using the Illumina TruSeq ver 2 kit and sequenced on an Illumina HiSeq2000. Raw metagenomic reads were uploaded to the MGRAST server (Meyer et al., 2008) under accession numbers: 4681149.3, 4680632.3, 4680631.3, 4680180.3, 4601511.3, and 4554870.3 and processed with a standard pipeline. Taxonomic assignments were performed using the GenBank database and annotation was conducted using Subsystems, KEGG, or IMG terms with a 1e- 5 e-value cutoff, min 60% identity, and min 15 aa alignment length. Sequences associated with functions responsible for heavy metal metabolism and sorption were identified in the IMG annotations using keywords and assigned taxonomically using the Workbench function and the Genbank database. Statistical analyses, unless separately stated, were performed using STAMP (Parks and Beiko, 2010), Fisher's exact test, Newcombe-Wilson confidence interval method, and Benjamini-Hochberg FDR correction.

Samples KOW_2 and ZS_3 were subjected to plasmid DNA isolation using PlasmidMidi kit (A&A Biotechnology, Gdansk). Obtained DNA was processed with PlasmidSafe exonuclease (Epicentre) to get rid of chromosomal contaminants and subjected to standard Illumina library preparation protocol. Sequencing reads were processed using MG-RAST (accession numbers: 4680178.3 and 4680633.3), as described above.

### 16S rRNA gene analysis

Metagenomic DNA purified from enrichment cultures was used as a template for 16S rRNA gene amplification as well as for metagenome sequencing. The 16S rRNA sequences were amplified using a method according to the Lundberg et al. (2013). 20 ng of DNA template was uniquely tagged with oligos BacV3V4F_tag (GTTCA GAGTTCTACAGTCCGACGATCNNNNNNNNAGCCTACGGGNGGCWGCAG) and BacV3V4R_tag (TTGGCACCCGAGAATTCCANNNNNGCGACTACHVGGGTATCTAATCC). After tagging, amplification was performed with primers RP1 (AATGATACGGCGACCACCGAGATCTACACGTTCAGAGTTCTACAGTCCGA), and RPI (CAAGCAGAAGACGGCATACGAGATNNNNNNGTGACTGGAGTTCCTTGGCACCCGAG AATTCCA). Amplicons were sequenced on MiSeq (Illumina) using paired-end technology, with 2 × 300 nt fragments length. Overlapping amplicon fragments were merged with flash (Magoc and Salzberg, 2011) using default parameters. Molecular tags and primer sequences were identified using pattern matching. Sequence fragment 5′ to the forward linker (forward tag) and the fragment 3′ to the reverse linker (reverse tag) were extracted and concatenated to form that sequence's molecular tag (MT), and the sequence occurring between the forward and reverse template–specific primers was extracted for analysis. Each unique MT observed in a sample was considered a unique MT category. For MT categories containing ≥2 sequences multiple alignment with MUSCLE (Edgar, 2004) was performed and consensus sequences were calculated. Sequences were processed with Uparse pipeline (Edgar, 2013). Briefly, barcodes were striped with fastq_strip_barcode_relabel2.py. Stripped sequences were quality filtered with usearch -fastq_filter -fastq_maxee 0.5 -fastq_trunclen 250. Reads were dereplicated using usearch -derep_fulllength -sizeout options and singleton sequences were removed. OTUs were clustered using -cluster_otus. Reads were then mapped back to OTUs using usearch globalreads.fa -dbotus.fa -strand plus -id 0.97 -ucmap.uc and otu_table was constructed using uc2otutab.py. Taxonomic assignment was done using RDP Classifier (Wang et al., 2007). Amplicon sequences where deposited in NCBI SRA (BioProject PRJNA327169) and the information about taxonomic assignments of 16S rRNA reads were presented in Table S1.

### Sorption

To examine sorption abilities, raw KOW and ZS microbial mats were dried at 80°C to a constant dry weight. Subsequently, 300–500 mg of dried samples were resuspended in 10 mL of solutions containing As(III), Cd(II), Co(II), Cu(II), and Fe(III) at a final concentration of 25 mM of each metal/metalloid, and pH 6.5. After 24 h of incubation at 22°C with shaking at 120 rpm, pellets were centrifuged (5 min, 10000 × G) and supernatants were collected and analyzed by ICP-MS.

### Desorption

To determine the strength of As, Cd, Co, Cu, and Fe binding by KOW and ZS microbial mats, the 300–500 mg samples of dried mats were resuspended in 10 mL of the following extraction buffers: (i) 0.1 M phosphate buffer, pH = 7.2 (exchangeable fraction); (ii) 0.05 M EDTA pH = 7 (mobile heavy metals fraction); (iii) 0.11 M acetic acid pH = 3.5 (heavy metals bound mainly to carbonate complexes); and (iv) deionized water (control and loosely bonded elements) (Antosiewicz et al., 2008). Metal extraction was performed by incubating the samples in buffer at 22°C for 24 h with shaking at 120 rpm, followed by analysis as described above (see Section Sorption).

### Scanning electron microscopy (SEM)

Freshly collected microbial mats were mechanically fragmented and placed on SEM pin stubs (6 mm), fixed, and dried in formaldehyde vapor. Samples were then analyzed with a Hitachi S-3400N scanning electron microscope at a voltage of 15 kV. For energy dispersive X-ray microanalysis (EDS), samples were examined at a voltage of 20 kV.

### Isolation and growth analysis of dissimilatory ${\text{AsO}}_{4}^{3-}$, Fe^3+^, and ${\text{So}}_{4}^{2-}$ reducing bacteria

Isolation of dissimilatory reducing bacteria using ${\text{AsO}}_{4}^{3-}$, Fe^3+^, or ${\text{SO}}_{4}^{2-}$ as the terminal electron acceptor was performed under anaerobic conditions [in 100 mL serum bottles with N_2_:CO_2_ (80:20) injected into the headspace] in MSM medium supplemented with 5 mM sodium lactate and 2.5 mM of proper salt of electron acceptor (MgSO_4_ for sulfate reducers and Na_2_AsO_4_ for arsenate reducers) (Drewniak et al., 2014) or modified FWA-Fe(III) citrate medium (C_6_H_5_FeO_7_ for iron reducers) (Lovley and Phillips, 1988). The prepared media were inoculated with KOW and ZS microbial mats 10:1 (v/v) and incubated for 7 days at 22°C with occasional mixing. Every 7 days, cultures were passaged by transfer to fresh medium at a ratio of 1:10 (v/v). After five passages, dissimilatory ${\text{AsO}}_{4}^{3-}$, ${\text{SO}}_{4}^{2-}$, and Fe^3+^ reduction abilities of the isolated consortia were tested in detail. The growth of ${\text{AsO}}_{4}^{3-}$, Fe^3+^, and ${\text{SO}}_{4}^{2-}$ reducing bacteria was analyzed using an epifluorescence microscope (Nikon Eclipse 80i) after staining with 4′,6-diamidino-2-phenylindole (DAPI). The resulting images were analyzed with NIS-Elements BR (Nikon) imaging software.

## Results

### Microbial mat chemistry

The microbial mats at the both locations (KOW and ZS) exist under similar and stable environmental conditions. Average water temperature measured during 3 years was stable at 10.2 and 10.5°C (for ZS and KOW, respectively). pH of the ZS mine water ranged from 7.59 to 8.01, while pH of the microbial mat ranged from 7.53 to 7.61. In contrast, pH of the KOW mine water was more acidic (5.83–6.0) and was similar to that of the microbial mat (5.67–5.70). In both mines, the heavy metal concentrations in water adjacent to the microbial mats was quite similar and, for most elements, below 1000 μg/L (Table 1). The only exception was arsenic concentration in the ZS water (5784 μg/L), which is one of the highest levels measured in surface water worldwide (Ravenscroft et al., 2009). In contrast to the mine water, extremely high concentrations of iron (5.8–6.2%), arsenic (~0.67–19.4%), aluminum (~10.4–14.5%), and manganese (0.1–7.7%) as well as high concentrations of uranium (152–195 mg/kg), lead (49–252 mg/kg), zinc (415–461 mg/kg), cobalt (15–111 mg/kg), copper (57–77 mg/kg), nickel (25–81 mg/kg), chromium (28–46 mg/kg), and vanadium (27–46 mg/kg) were present in both dried microbial mats (Table 1). These results indicate a huge capacity of both microbial mats to accumulate heavy metals and act as a specific bio-filter of mine dewatering systems.

**Table 1:** **Heavy metal content in microbial mats and mine water in Kowary uranium mine and Zloty Stok gold mine**.

			**As**	**U**	**Fe**	**Al**	**Mn**	**Cu**	**Co**	**Cd**	**Ni**	**Zn**	**Pb**	**Mo**	**Cr**	**V**
KOW	Mine water	μg·L^−1^	80	352	64.62	3.17	326	0.39	0.92	–	3.47	13.08	0.07	3.00	0.136	1.59
	Microbial mats	mg·L^−1^	6746	195	58200	10400	1039	57	15	–	25	415	49	3	28	27
ZS	Mine water	μg·L^−1^	5784	8.9	66.88	2.34	47.11	1.07	0.51	–	0.33	11.07	0.34	8.99	0.08	2.6
	Microbial mats	mg·L^−1^	19427	152	62400	14550	7733	77	111	4	81	461	252	10	46	46

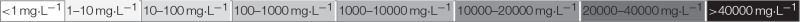

### Morphology of the microbial mats and EDS analysis

**Figure 1:**
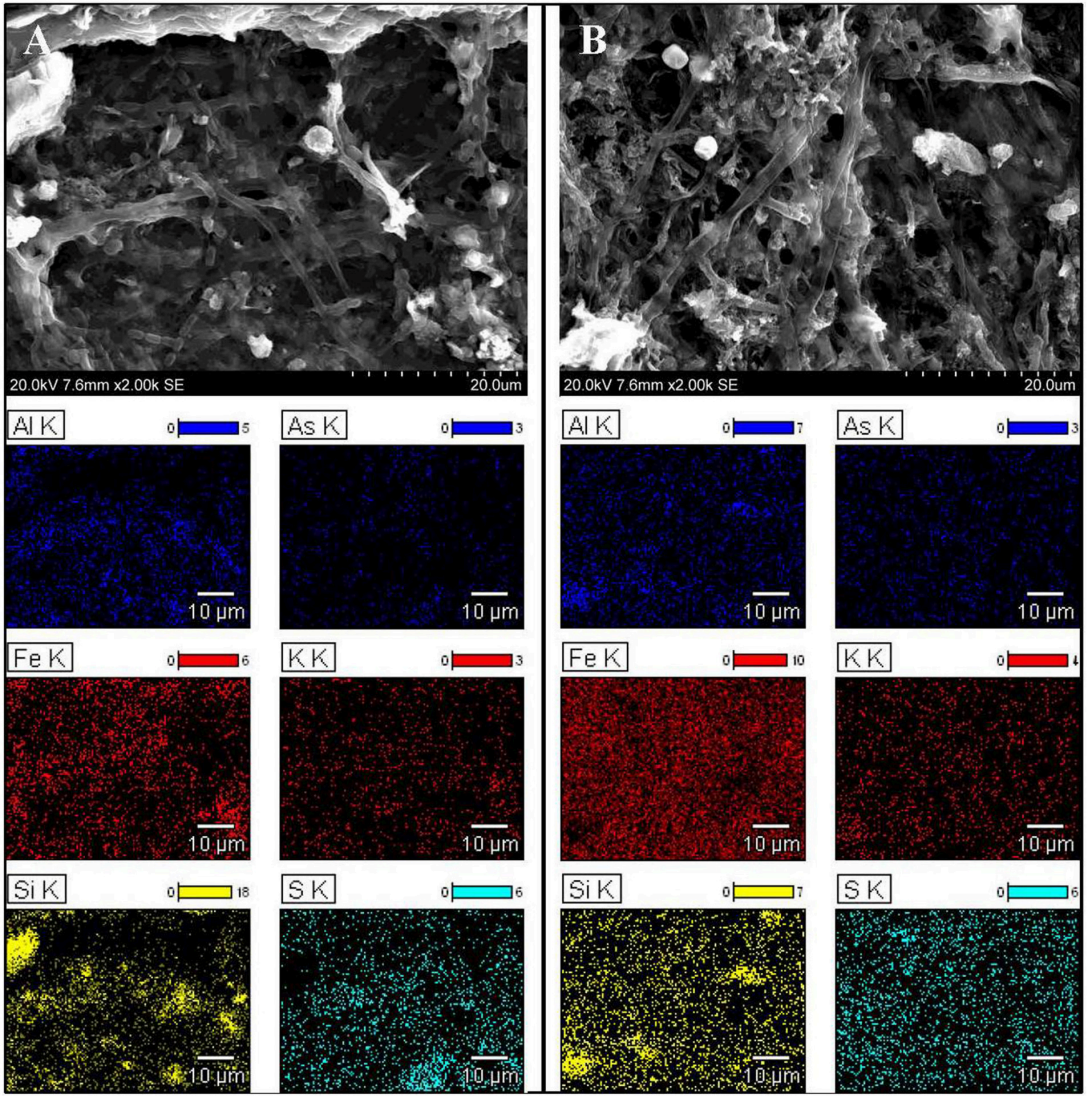
**Morphology and distribution of chosen elements in microbial mats**. Scanning electron micrographs and energy-dispersive X-ray spectroscopy results showing the structure and distribution of chosen elements in microbial mats from **(A)** ZS and **(B)** KOW.

To obtain further detail about heavy metal filtration by microbial mats, it was necessary to analyze their structure and determine whether heavy metals accumulate inside of bacteria cells or in the extracellular matrix. SEM confirmed a typical multilayered, heterogeneous mosaic structure containing filamentous, cocci, and cylindrical morphotypes of microorganisms without significant domination of any particular form (Figure 1). Filamentous bacteria formed the backbone of the mats, wherein bacteria with other morphotypes were interleaved by extracellular matrix. EDS comparative sample surface microanalysis showed that the most abundant metals were aluminum, iron, and arsenic. Metals were found dispersed over the entire surface of the mats as well as in the bacterial cell walls and extracellular matrix (Figure 1). However, the distribution of metals and metalloids within the microbial mat structure was different between the KOW and ZS samples. Iron abundance at the surface of the KOW mat reached 56.53 wt%, while in the ZS samples it was only 17.75 wt%. The concentration of aluminum at all analyzed points of the KOW mat was ~2.5%, which was approximately four times lower than aluminum levels observed in the ZS mat. Interestingly, the EDS analysis showed that the arsenic concentration in the KOW mat surface structure reached ~15 wt%, which was 6–10% higher than arsenic levels observed in the ZS mat. Moreover, the ICP-MS analysis indicated that the total arsenic concentration was three times higher in the ZS mat compared to the KOW mat (19427 and 6746 mg/kg d.w., respectively). Significant differences in the total concentration of metals in both mats were also observed for Mn, Co, Ni, Pb, and Mo (Table 1). Importantly, the observed differences cannot simply be explained by differences in the metal concentrations in mine water, since the concentrations of the elements in the mats did not closely correlate with levels in the water (Table 1).

### Capacity of microbial mats for sorption of heavy metals

**Figure 2:**
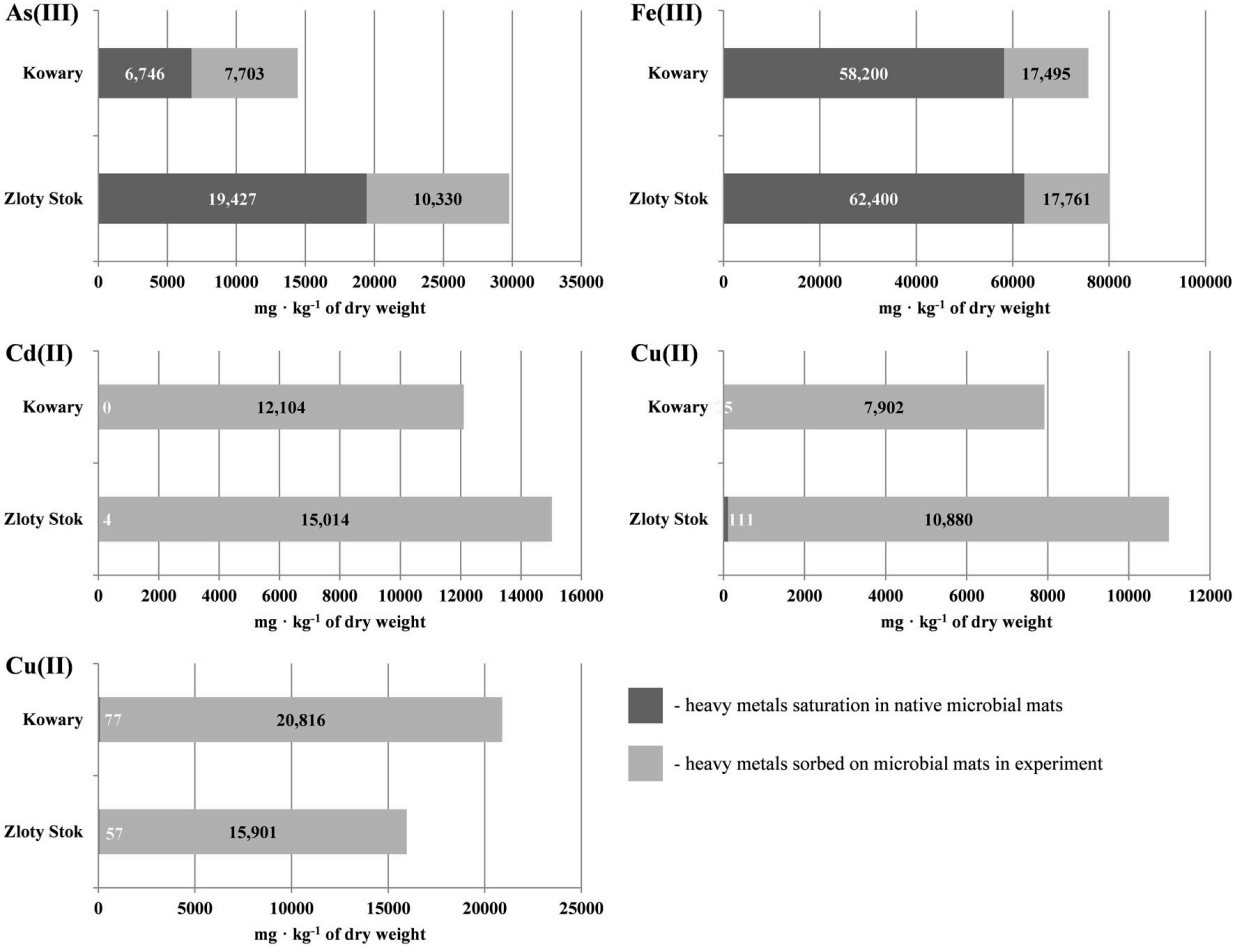
**Sorption capacity of microbial mats from KOW and ZS for As(III), Cd(II), Co(II), Cu(II), and Fe(III)**.

**Figure 3:**
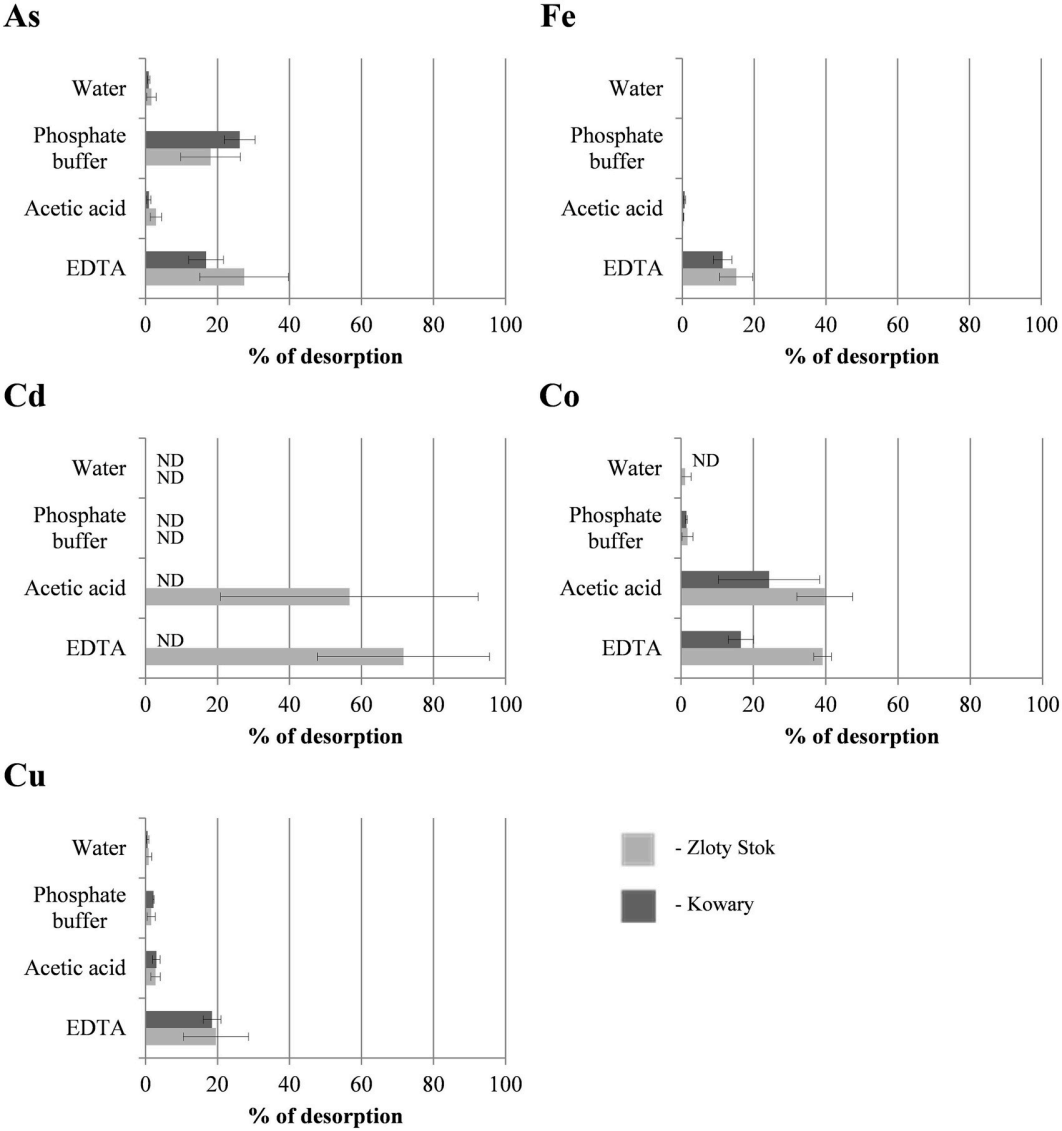
**Extraction efficiency of As, Cd, Co, Cu, and Fe accumulated in microbial mats from KOW and ZS**. Four different eluents were used: deionized water, 0.1 M phosphate buffer, pH = 7.2, 0.11 M acetic acid pH = 3.5, and 0.05 M EDTA pH = 7. Each bar shows the average amount of metal ion extracted from the mat (3 repeats, SD showed on graph). ND, metal not detected in the extracted solution.

Sorption experiments with the dried biomasses were next performed to determine whether the microbial mats were saturated with heavy metals. All sorption experiments were performed at pH 6.5 to avoid uncontrolled precipitation of metals and we found that the mats were not completely saturated with As, Cd, Cu, Co, and Fe as additional amounts of metal ions could be adsorbed (Figure 2; for As and Fe the saturation reached 46.7 and 76.8%, respectively, while for the other metals the saturation ranged from 0.0 to 1.0%). Level of sorption refers to the individual elements and depends on the initial saturation of the mats. The highest saturation of the raw mats was observed for iron, and reached 76.8% in the KOW and 77.8% in ZS samples, and of this metal sorption capacity was measured (58200 and 62400 mg/kg d.w., respectively). Arsenic concentration in raw ZS microbial mat was 19427 mg/kg d.w. (Table 1), and the sorption experiment showed that the mat could still absorb an additional 10330 mg of As/kg d.w., which represents 53.2% of the initial value (Table 1). In contrast, the initial arsenic concentration in KOW mat was 6746 mg/kg d.w., and the sorption capacity reached 14449 mg/kg d.w. On the other hand, the concentration of cadmium, cobalt, and copper in the raw mats was very low. The potential sorption capacity of both mats is shown on Figure 2. The ZS microbial mat exhibited significantly higher sorption capacity for As, Fe, Cd, and Co than the KOW mat. Surprisingly, sorption of Cu to the KOW mat was almost 25% higher than that observed for ZS. Despite these differences both microbial mats show potential as a valuable sorption material. Moreover, the importance of the microbial mats as a specific filtration system for heavy metals was supported by the desorption experiment. Our analysis showed that most of the absorbed elements could only be released to the mobile fraction in presence of strong extractants (Figure 3). EDTA was the most effective and the only eluent that readily contributed to release of each of the examined elements. Desorption yield of cadmium by EDTA exceeded 50%, whereas yield for all other metals was ~20–40%. Acetic acid bound mainly to carbonate complexes and was able to elute significant amounts of cobalt and cadmium (up to 40 and 56.64%, respectively). In contrast, phosphate buffer was only effective for extraction of arsenic (up to 30%; Figure 3), while, in presence of MiliQ water only 5% of metal ion recovery was observed. Notably, mat from the ZS mine exhibited much higher sorption and desorption capacity than mat from the KOW, what suggest that observed water purification process is based on different mechanisms.

### Metagenomic analysis of metabolic potential for heavy metal removal (stabilization)

It is well-known that sorption is one mechanisms responsible for heavy metal removal. This process is independent of metabolic pathways, but can be enhanced by the metabolic activity of the entire bacterial community. Therefore, it is important to determine if and how metabolism is involved in heavy metal sorption. To answer this question, we next performed a metagenomic analysis that focused on the pathways connected with heavy metal sorption, precipitation, and stabilization.

#### Phylogenetic diversity of microbial mats

**Figure 4:**
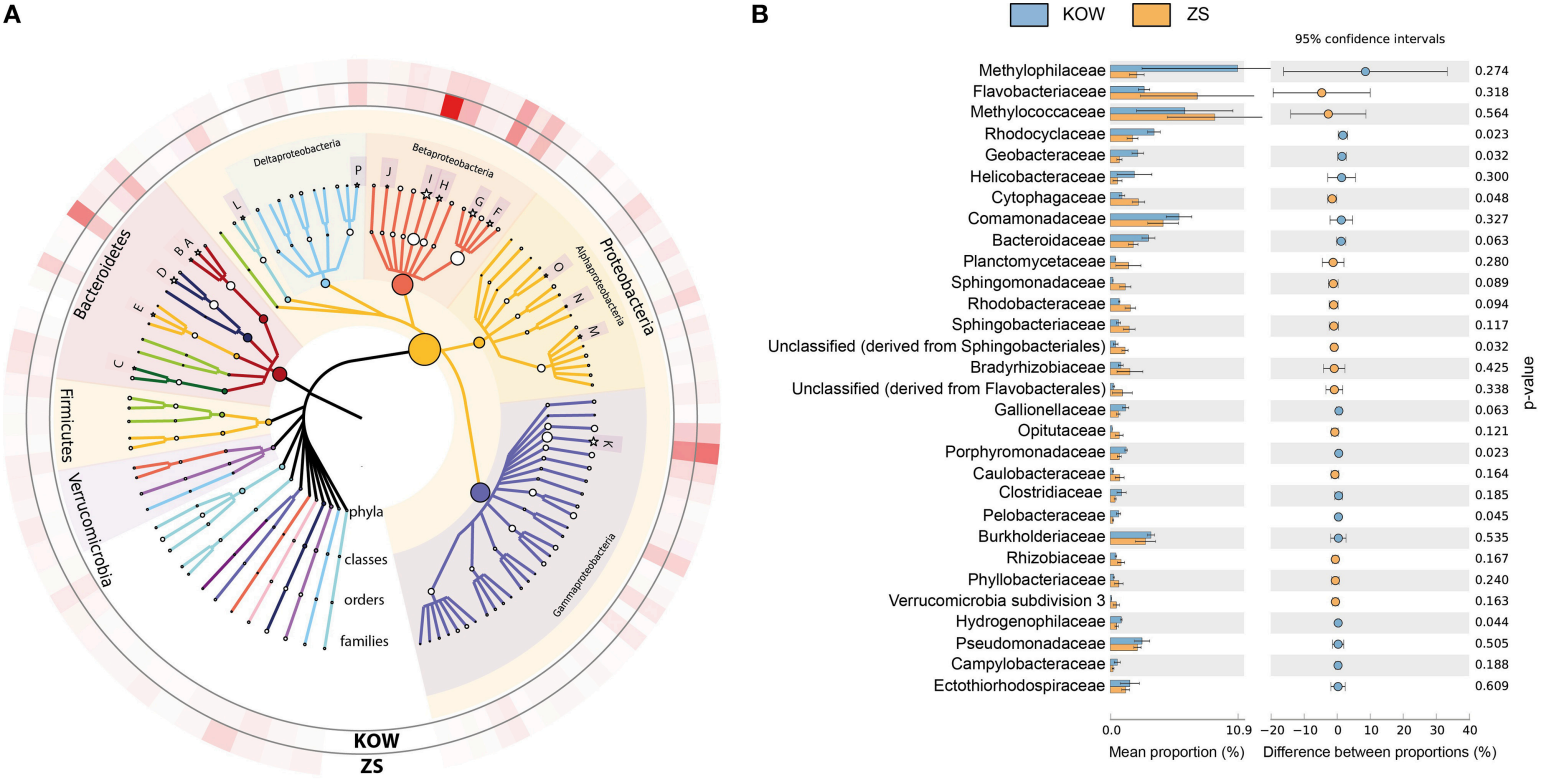
**Most abundant families found in the analyzed microbial mats. (A)** Phylogenetic tree of the most abundant families identified in analyzed metagenomes. The size of tree node represents the abundance of a particular taxon in the analyzed metagenomes. Families with significant differences between analyzed metagenomes are marked with a star. On the outer ring, family abundances are showed as a heatmap. A, *Bacteroidaceae*; B, *Porphyromonadaceae*; C, *Sphingobacteriaceae*; D, *Flavobacteriaceae*; E, *Cytophagaceae*; F, *Burkholderiaceae*; G, *Comamonadaceae*; H, *Rhodocyclaceae*; I, *Methylophilaceae*; J, *Hydrogenophilaceae*; K, *Methylococcaceae*; L, *Helicobacteraceae*; M, *Bradyrhizobiaceae*; N, *Sphingomonadaceae*; O, *Rhodobacteraceae*; P, *Geobacteraceae* **(B)** Families differing in abundance between analyzed communities. *p*-values calculated in STAMP using Welch's test, with no correction used.

To determine the microbial diversity within the mats shotgun metagenomic analysis was used. M5NR-based taxonomic annotation using MG-RAST revealed high similarity between both environments. Statistical analysis did not show any significant differences between observed communities, however we noticed some minor dissimilarities between samples. *Methylophilaceae* and *Methylococcaceae* were the most abundant families in both samples (6.390 ± 4.132 and 8.970 ± 4.086% for *Methylococcaeae* and 10.930 ± 8.217 and 2.272 ± 0.626% for *Merthylophilaceae* in KOW and ZS, respectively), but with high fluctuations throughout the samples (Figures 4A,B). In the KOW community we noticed an enrichment of the *Epsilonproteobacteria* class, mainly of the *Helicobacteraceae* family, including *Sulfuricurvum* and *Sulfurimonas* genera. On the other hand, the ZS community was more abundant with *Alphaproteobacteria*, mainly *Sphingomonadaceae, Rhodobacteraceae*, and *Bradyrhizobiaceae* families (Figure 4B). A minor difference in the community structure was also observed at the species/genus level. Among filamentous bacteria, which form the backbone of the mats (as shown by SEM and EDS analysis) the following genera's dominated and had similar distribution in both samples: *Leptothrix, Thiothrix, Beggiatoa*, and *Halothermothrix* (Table S2).

#### Metabolic diversity and potential for heavy metal removal

To determine if bacterial metabolism plays a role in the transformation of heavy metals, data from metagenomics DNA sequencing were annotated using MGRAST against KEGG and SEED databases. The metagenomic analysis took into account the overall metabolic capacity as well as the specific groups of genes encoding proteins directly or indirectly involved in heavy metal transformation. Metagenomes of both microbial mats showed a similar distribution of genes representing the major metabolic pathways (Table S3). Minor differences were observed in genes involved in amino acid metabolism-related functions, especially in the branched-chain amino acids subsystem, which were more abundant in the ZS metagenome (2.276 ± 0.277%) than in KOW samples (1.859 ± 0.059%). Enrichment of this group of genes suggests a higher concentration of proteins in the ZS mine environment, which may make it easier for the community to use it as a source of nitrogen. This observation is supported by the fact that in KOW metagenomic DNA, the number of genes from the nitrogen fixation subsystem were elevated and reach 0.345 ± 0.033%, whereas ZS only contained 0.172 ± 0.051%. In the KOW samples there was a higher number of electron donating subsystem sequences compared to the ZS samples (1.226 ± 0.083 vs. 1.035 ± 0.047%, respectively), while more genes from the ZS samples were annotated as an electron accepting reaction subsystem than in KOW (0.740 ± 0.042 vs. 0.718 ± 0.046%, respectively). All subsystem annotation statistics are available in Table S4.

##### Biofilm formation-related genes

It is well-known that biofilm structure readily affects sorption capabilities of the microbial communities (Malik, 2004), and therefore we next assessed the genes involved in biofilm formation and extracellular polysaccharide production. The pool of reads annotated to genes involved in the mentioned processes exceeds 2% of all genes identified in both metagenomes, and the taxonomic distribution of those genes resembled the community structure described in the taxonomic analysis (Supplementary Figure S2).

The analyzed microbial mats did not significantly differ in terms of capsular and extracellular polysaccharide subsystems (1.440 ± 0.099 and 1.560 ± 0.223% in KOW and ZS, respectively), where *Proteobacteria*: *Burkholderiales* and *Pseudomonadales*, as well as *Flavobacteriales* dominated over other orders (Supplementary Figure S2). There was also no significant difference in the abundance of genes involved in quorum sensing, biofilm formation, adhesion, or flagella and motility; however, there were differences in the taxonomical distribution among these functions. In both metagenomes, *Methylococcales* had a similar contribution to all biofilm-related functions, whereas *Methylophilales* and *Campylobacterales* were more prominent in KOW and *Flavobacterales* was more prominent in the ZS metagenome (Supplementary Figure S2).

##### Heavy metal homeostasis

**Figure 5:**
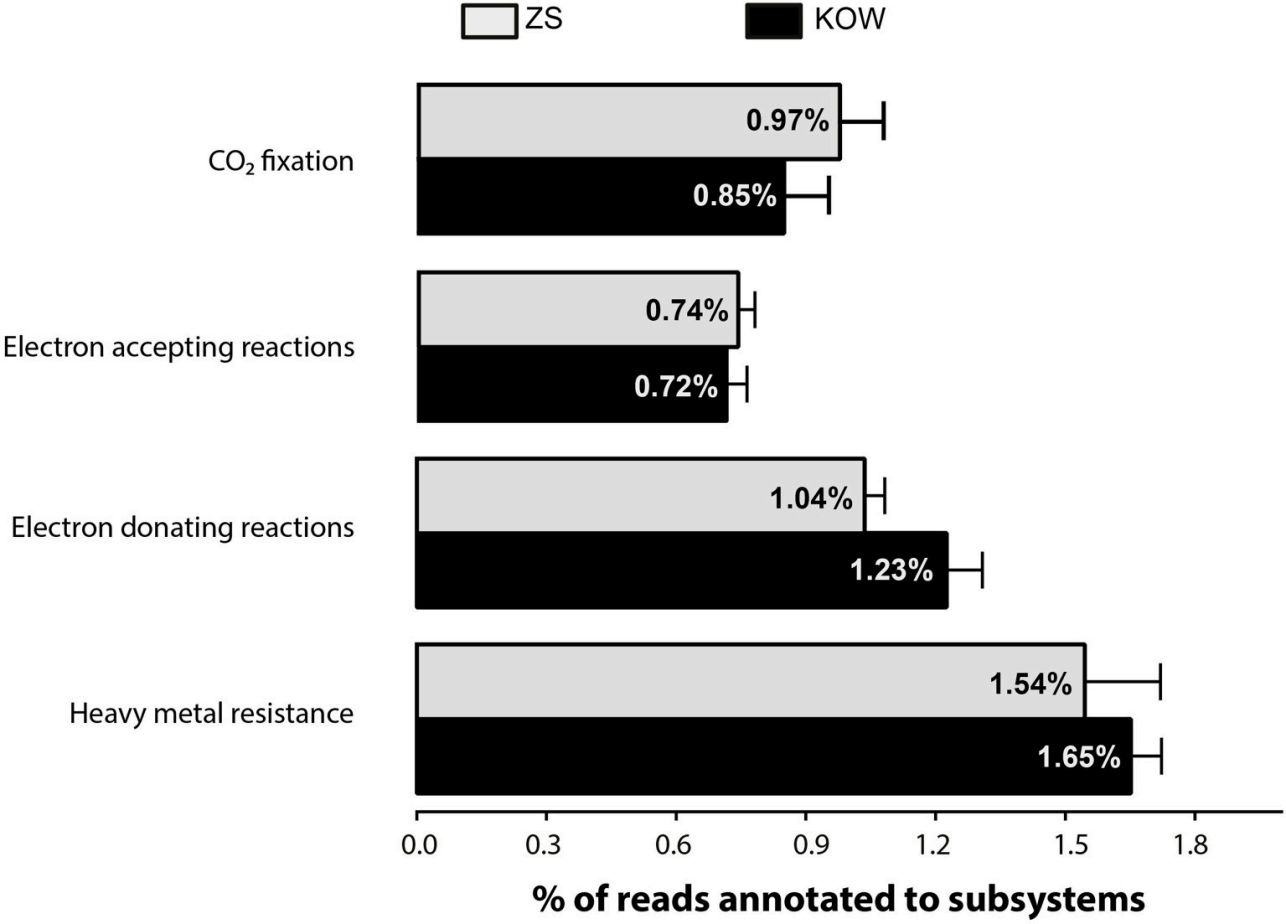
**Abundance of subsystems involved in heavy metal homeostasis among all subsystem-annotated metagenomic reads**. Heavy metal resistance includes the following subsystems: arsenic resistance, resistance to chromium compounds, zinc resistance, cadmium resistance, cobalt-zinc-cadmium resistance, copper homeostasis, mercuric reductase, mercuric resistance operon, and tellurite resistance: chromosomal determinants.

In accordance with bacterial needs, heavy metals can be transformed (e.g., oxidized, reduced, methylated, or complexed) and used as a source of energy, terminal electron acceptors, or enzyme structural elements. It is also known that metals taken up from the environment are often excreted outside the cell (in a less toxic form) by various detoxification systems (Silver and Phung Le, 2005). Thus, to understand the potential of the microbial mats to transform heavy metals, we determined which types of transformation processes dominate within the analyzed metagenomes. Moreover we determined also distribution of genes from transformation pathways among members of taxonomic group, to decipher potential role of different microorganisms in heavy metal homeostasis. Scrutiny of the subsystem-based annotations of metagenomes from ZS and KOW allowed us to highlight groups of functions potentially involved in heavy metals homeostasis as follows: (i) heavy metal resistance; (ii) electron accepting reactions possibly linked with direct and indirect metal and metalloid reduction (mostly respiratory processes); (iii) CO_2_ fixation and electron donating reactions, connected with chemolithotrophs known to assimilate CO_2_, and draw energy from oxidation of inorganic compounds (e.g., mineral deposits or soluble compounds); and (iv) subsystems involved in heavy metals complexation (mainly siderophores). A comparison of the representation of functions showed that functional groups were equally represented in both communities (Figure 5). This general comparison suggests that microbial mat communities are well-balanced in terms of microorganism abundance, especially those responsible for heavy metal mobilization, and immobilization.

**Figure 6:**
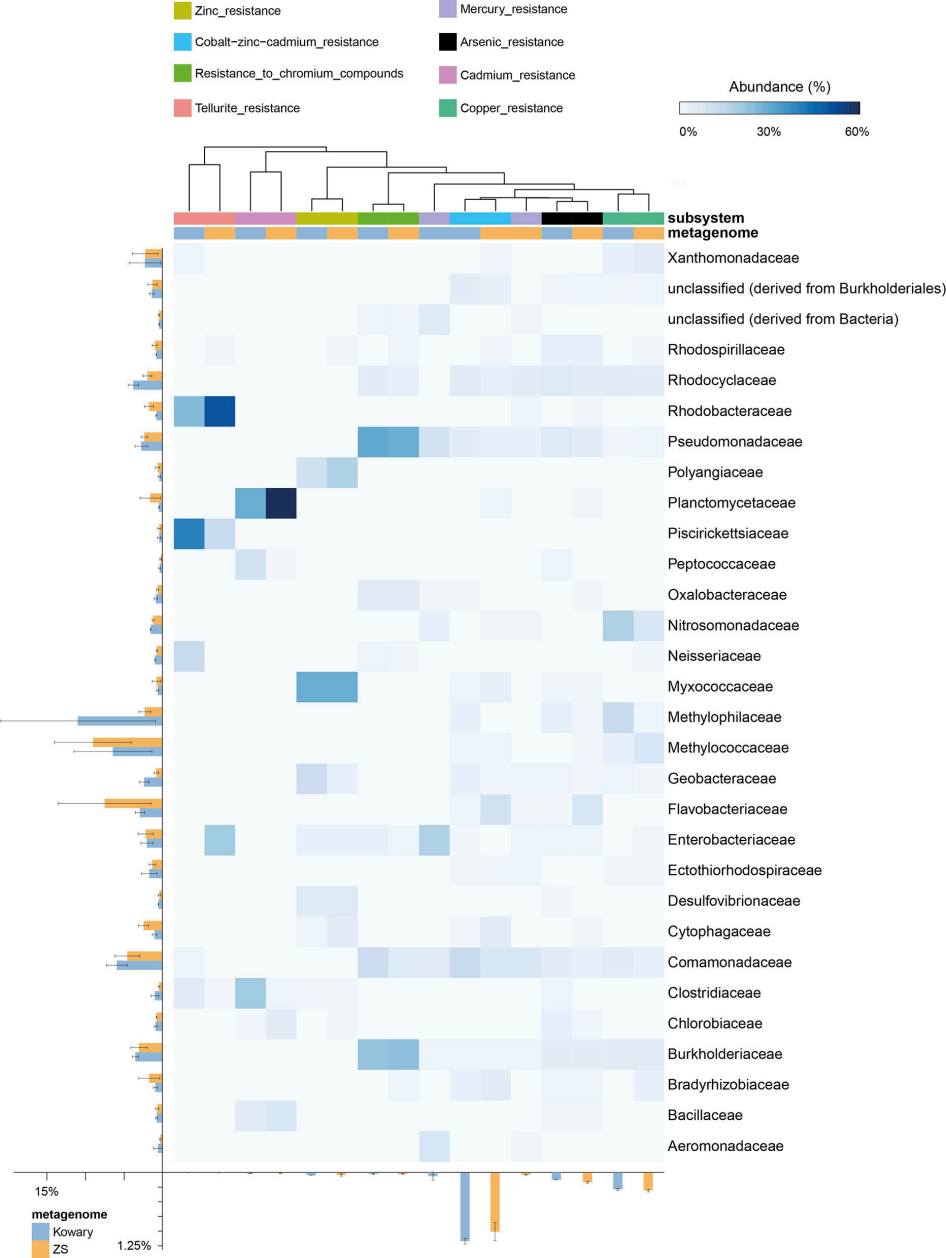
**Taxonomic distribution of reads assigned to heavy metal resistance subsystems**. The heatmap shows relative abundance of reads classified to a given family in a particular subsystem. Total abundances in metagenomes are shown for families and subsystems on the left and at the bottom, respectively. Subsystems were clustered using the average neighbor (UPGMA) method. Mercury resistance indicates summarized data for mercuric reductase and mercury resistance operon subsystems.

Heavy metal resistance (HMR) genes include those mediating arsenic, copper, cadmium, chromium, cobalt-zinc-cadmium (CZC), mercury, silver, tellurium, and zinc resistance. However, the highest abundance of HMR genes is mainly due to the presence of Cd, Zn, and Co detoxification systems. In both metagenomes, CZC resistance genes coded for over 95% of the putative proteins engaged in HMR, which accounts for more than 1.1% of all metagenomic reads (Figure 5). Taxonomic distribution within the CZC resistance subsystem was evenly divided between seven dominating families: *Comamonadaceae, Rhodocylaceae, Cytophagaceae, Methylococcaceae, Myxococcaceae, Burkholderiaceae*, and *Flavobacteriaceae* (Figure 6). A similar taxonomic pattern was observed for arsenic resistance genes, which represented the third largest group of the HMR functions (Figure 6). In both metagenomes, arsenic resistance genes were assigned mainly to *Comamonadaceae, Rhodocylaceae, Rhodospirillaceae, Burkholderiaceae, Pseudomonadaceae, Flavobacteriaceae, Enterobacteriaceae*, and *Geobacteraceae.* These families were also found in the dominant groups of other HMR subsystems; however, their distribution was more diverse and irregular. The vast majority of zinc resistance genes were assigned to *Myxococcaceae, Polyangiaceae, and Geobacteraceae* for the KOW community and to *Myxococcaceae, Polyangiaceae*, and *Cytophagaceae* within the ZS microbial mat. In addition, the *Burkholderiaceae, and Pseudomonadaceae* families dominated in chromium resistance, representatives of *Enterobacteriaceae* were the most abundant in mercury detoxification systems, and *Methylococcaceae, Methylophilaceae, and Nitrosomonadaceae* were predominant in copper tolerance (Figure 6).

Both microbial mat metagenomes contained a number of genes annotated to be involved in electron-accepting reactions, and therefore potentially associated with metal and metalloid reduction. Among these subsystems, the most abundant genes encoded for proteins similar to terminal cytochrome c oxidases (Supplementary Figure S3), representing 0.198 ± 0.009 and 0.285 ± 0.062% of all metagenome reads for KOW and ZS, respectively. However, cytochrome c is a heterogeneous family of proteins known for their ability to use oxidized species of Fe(III), Cr(VI), and Mn(IV) as terminal electron acceptors, but also engage in respiration processes utilizing an enormous number of organic compounds. Therefore, an abundance of cytochrome c-like genes may serve as an indirect marker for metal reduction, which is consistent with the results of the taxonomic distribution analysis. In both microbial mats, cytochrome c sequences closely related to methanotrophic genera were more abundant than those observed in heavy metal transforming microorganisms. Cytochrome c sequences from ZS were assigned to *Flavobacterium, Methylococcus*, and *Burkholderia*, whereas those from the KOW community belonged mainly to the *Methylotenera, Methylobacillus, and Geobacter* genera (Table S5). In addition, various representatives of *Comamonadaceae, Pseudomonadaceae, Methylococcaceae, Methylophilaceae, Flavobacteriaceae, Rhodocyclaceae, Nitrosomonadaceae*, and *Geobacteraceae* were found in both metagenomes (Table S5).

In addition to cytochromes, dissimilatory sulfate reductases are representatives of another group of electron acceptors known to indirectly engage in metal precipitation through sulfide production. Nitrate and nitrite reductase as well as nitrogenases have also been previously described to contribute indirectly in heavy metal precipitation. These enzymes participate in the processes by elevating the pH through ammonia production, what in turn triggers precipitation of the metal hydroxides. The nitrate and nitrite ammonification subsystem contributed to 0.351 ± 0.065 and 0.298 ± 0.062% of all reads annotated to subsystems in KOW and ZS and was mainly assigned to *Comamonadaceae, Hydrogenophilaceae, Alteromonadaceae, Burkholderiaceae*, and *Shewanellaceae*. In addition, nitrogenase-related genes contributed to 0.310 ± 0.033 and 0.102 ± 0.051% of all reads in the KOW and ZS communities, respectively (Table S4), with *Pseudomonas, Dechloromonas*, and *Azotobacter* being the representative generas.

The fourth group of functions potentially involved in heavy metal homeostasis was previously observed in mineral oxidizing chemolithotrophs. These processes allow bacteria to grow autotrophically by fixing CO_2_ from the atmosphere. Energy production occurs through the use of inorganic compounds (mostly ferrous iron or reduced sulfur) as electron donors and oxygen as the final electron acceptor. Abundance analysis of the CO_2_ assimilation subsystem revealed that the most prevalent mechanism for CO_2_ binding was connected with ribulose-1,5-bisphosphate carboxylase (RuBisCo; 0.015 ± 0.005% in KOW and 0.007 ± 0.003% in ZS) (Supplementary Figure S4). RuBisCo is mainly derived from the *Thiobacillus* and *Acidithiobacillus* genera, both of which were present in KOW and ZS. However, we only assigned CO_2_ assimilation functions to mineral-oxidizing chemolithotrophs if they were associated with inorganic electron-donating reactions. A detailed analysis of the electron-donating reaction subsystems showed that several functions were associated with mineral-oxidizing chemolithotrophs, including: (i) Fe(II) oxidation (such as cytochrome c and multiple copper oxidases); (ii) oxidation of reduced inorganic sulfur compounds (sulfur oxidase and dehydrogenase, sulfite oxidoreductase and thiosulfate oxidase); and (iii) arsenite oxidation. The remaining functions, which constituted the majority of the reads within this subsystem, were assigned to organic compounds (Supplementary Figure S5).

Analysis of functions related to complexation of heavy metals revealed genes encoding enzymes that participate in the synthesis of organic acids (e.g., citrate synthase, aconitate hydratase) as well as organic ligands such as siderophores. From these functions, the latter were the most abundant (nearly 0.05% of all metagenomic reads) and highly diverse (Supplementary Figure S6). Pyoveridine siderophores were the most abundant siderophore subsystems (95%) (e.g., pyochelin, enterobactin, aerobactin, bacillibactin, and yersiniabactin), while *Pseudomonadace* and *Enterobacteriaceae* prevailed among the siderophore-annotated reads (Supplementary Figure S6).

Functions described above were also described in the context of mobile elements present in both environments. Both taxonomic and functional distributions of plasmid-source reads reflected those of whole metagenomes. However, ZS plasmid sample was enriched in *Alphaproteobacteria* and *Actinobacteria* annotations. Functional analysis revealed that both plasmid samples have more functions connected with transport, signaling, and processing of carbon and nitrogen sources (amino-acids, fatty acids, aromatic compounds). We did not observed any significant enrichment of heavy-metal resistance functions on plasmid sequences.

### Selection and characterization of dissimilatory reducing bacteria

**Figure 7:**
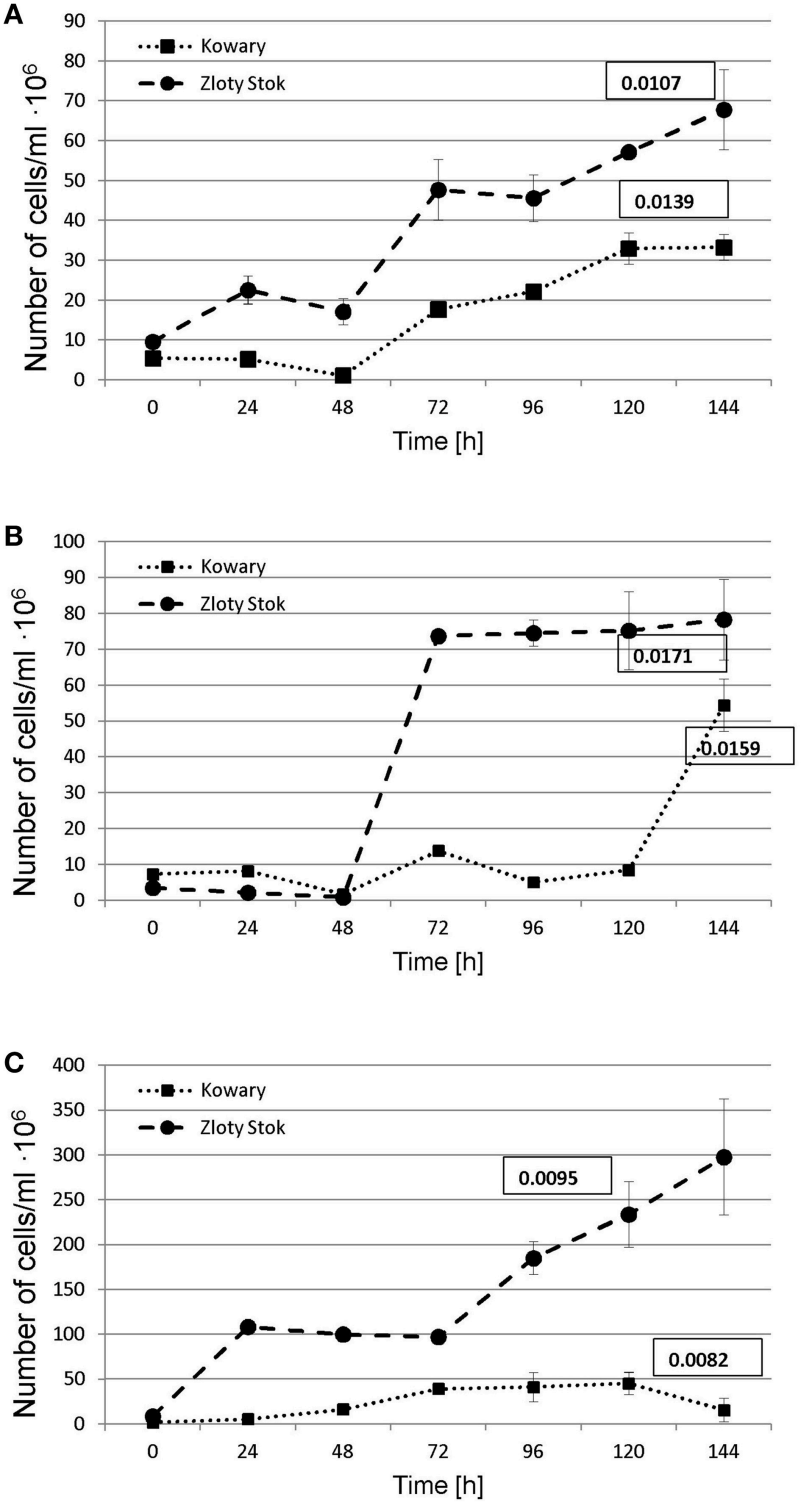
**Growth of dissimilatory arsenate (A), sulphate (B), and iron (Fe III) (C) reducers under anaerobic conditions on minimal salt medium supplemented with 5 mM sodium lactate and 2.5 mM of proper salt of electron acceptor (${\text{AsO}}_{4}^{3-}$, ${\text{SO}}_{4}^{2-}$, or Fe^3+^)**. Growth rates (number of doublings that occur per hour) are shown in the square.

**Figure 8:**
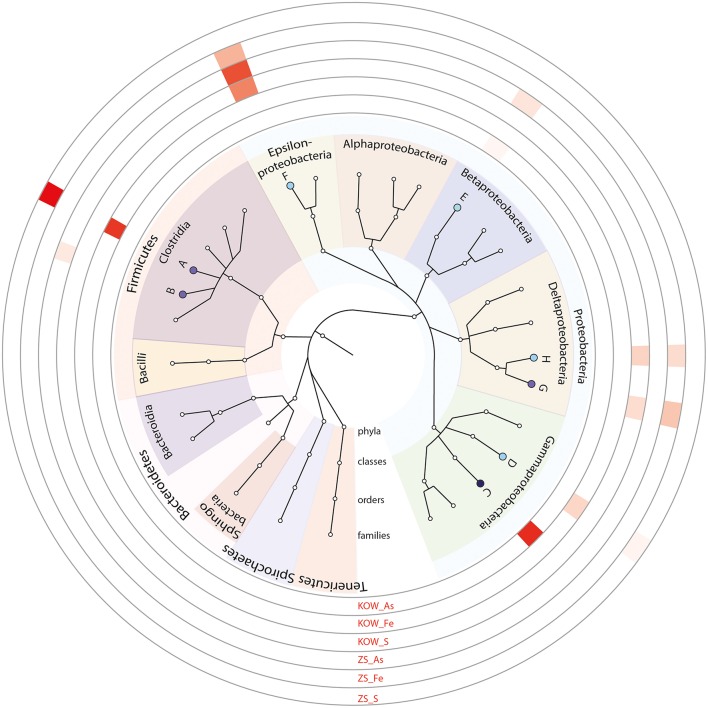
**Most abundant genera found in consortia using 16S rRNA amplicon sequencing**. On the outer ring, family abundances in individual consortia are showed as a heatmap. A, *Lachnospiraceae*; B, *Eubacteriaceae*; C, *Shewanellaceae*; D, *Enterobacteriaceae*; E, *Rhodocyclaceae*; F, *Campylobacteraceae*; G, Desulfomicrobiaceae; H, *Desulfovibrionaceae*.

A functional analysis of the microbial mats was also performed to complement the metagenomic approach, including the selection and characterization of microorganisms that were directly or indirectly involved in heavy metal immobilization. As immobilization of metals is usually connected with presence of anaerobic bacteria, three physiological groups of anaerobic microorganisms were selected based on their role in biogeochemical metal cycles: (i) sulfate-reducing bacteria—involved in heavy metal precipitation through sulfide production; (ii) iron-reducing bacteria—responsible for immobilization of iron and other metals (e.g., U, Cr); and (iii) dissimilatory arsenate reducing bacteria—arsenite producers having a high ability to precipitate with sulfides. For the selected groups of anaerobic microorganisms, the same method of isolation described in the Materials and Methods was used. After selection of stable, anaerobic respiratory consortia, the following parameters were investigated: growth kinetics (Figure 7), rate of terminal electron acceptor utilization (${\text{AsO}}_{4}^{3-}$, Fe^3+^, and ${\text{SO}}_{4}^{2-}$; Table 2), and bacterial diversity (Figure 8).

**Table 2:** **Growth parameters of selected microbial consortia during dissimilatory reduction of arsenate, sulfate, and iron**.

	**ZS_As**	**ZS_S**	**ZS_Fe**	**KOW_As**	**KOW_S**	**KOW_Fe**
**Microbial consortia**	**ZS**			**KOW**		
**Terminal electron acceptor**	**${\text{AsO}}_{4}^{3-}$**	**${\text{SO}}_{4}^{2-}$**	**Fe^3+^**	**${\text{AsO}}_{4}^{3-}$**	**${\text{SO}}_{4}^{2-}$**	**Fe^3+^**
^*^Number of cells/ml · 10^6^	57.2	7.8	233.8	33.0	54.4	45.4
Growth Rate [number of doublings • h^−1^]	0.0107	0.0171	0.0095	0.0139	0.0159	0.0082
Doubling time [h]	64.80	73.26	40.47	49.83	84.12	43.58
Time required for complete reduction [h]	168	>336	144	72	>336	144
Reduction rate [mg • L^−1^ • h^−1^]	2.232	0.786	1.944	5.208	1.012	1.944

* The maximum number of cells per ml in the logarithmic growth.

In all sets of experiments, anaerobic growth of the microbial mats and utilization of terminal electron acceptors was observed; however, only sodium arsenate was entirely utilized. The ZS_As consortium completely reduced As(V) to As(III) after 168 h with an average reduction rate of 2.232 mg As(V) • L^−1^ • h^−1^, whereas the reaction catalyzed by the KOW_As community was two times faster (72 h) with a reduction rate of 5.208 mg As(V) • L^−1^ • h^−1^ (Table 2). The growth rate (number of doublings that occur per hour) of the KOW_As community was also higher than that of the ZS_As (0.0139 h^−1^ and 0.0107 h^−1^ respectively), despite the fact that the ZS community reached a higher cell density per mL (57.2 • 10^6^ for ZS_As vs. 33 • 10^6^ for KOW_As samples; Table 2). Dissimilatory Fe(III) reduction was much slower than for the arsenate. In both microbial mats, the maximum efficiency of iron reduction was reached after 144 h with an average reduction rate of 1.944 mg • L^−1^ • h^−1^ (Table 2). The observed growth rate for the ZS_Fe consortium was higher than that for the KOW_Fe samples (0.0095 h and 0.0082 h, respectively). Notably, the cell density of the ZS_Fe culture (233.8 • 10^6^ cfu/ml) was five times greater than the cell density of the KOW_Fe by (45.4 •10^6^ cfu/ml; Figure 7), thus suggesting a much longer lag phase. The lowest reduction efficiency was observed for sulfate as an electron acceptor. In both microbial mat cultures, we observed a 10% decrease in sulfate concentration after 144 h of incubation. After 7 additional days of culture, the sulfate concentration dropped to ~50% of the initial level and the reduction rate attained was 0.786 mg ${\text{SO}}_{4}^{2-}$ • L^−1^ • h^−1^ for ZS_S and 1.012 mg ${\text{SO}}_{4}^{2-}$ • L^−1^ • h^−1^ for the KOW_S community (Table 2). Surprisingly, the low reduction rate of sulfate did not affect the growth rate of either microbial mat, which was in contrast to our expectations. Under these conditions the consortia reached the highest observed growth rates (0.0171 h for ZS_S and 0.0159 h for KOW_S).

To obtain more information about the microorganisms from the isolated consortia, 16S rRNA gene fragments (region V3–V4; positions 341–785) were amplified, sequenced, and analyzed (Supplementary Materials). Biodiversity analyses of the consortia selected as dissimilatory reducers of iron, arsenic, and sulfate revealed that they were formed by highly specialized groups of microorganisms representing different phyla of bacteria. The sulfur reducers found in the ZS_S and KOW_S samples were similar and represented *Sulfurospirillum, Desulfomicrobium, Desulfovibrio*, and *Sulfuricurvum* genera (Figure 8). The *Lachnospiraceae* was the most abundant family in the KOW_Fe and ZS_Fe consortia; however, *Raoultella* was specific for KOW_Fe and *Yersinia, Bacteroides, Salmonella*, and *Aeromonas* were specific for ZS_Fe. Dissimilatory arsenate reduction seems to be driven by different communities in the KOW and ZS mats. In KOW_As the most abundant organism was found to be *Shewanella*, which is almost absent in ZS_As. Moreover, *Campylobacteraceae* (mainly *Sulfurospirillum)* was the most abundant family in ZS_As, with *Quatrionicoccus* and *Acetobacterium* having a 2-fold lower abundance (Figure 8).

## Discussion

Extreme environments are a perfect model to illustrate the potential and range of adaptive capacity of the microorganisms. In this study we have described naturally occurring microbial mats from the KOW and the Zloty Stok gold mine and analyzed the components in the context of the metabolic functions responsible for heavy metal homeostasis and water purification properties.

Bacteria present in mats primarily use easily assimilable nutrients; however, in harsh environment conditions, they are able to utilize various toxic organic compounds, radionuclides, and oxyanions as well as sequester heavy metals or metalloids (Bender and Phillips, 2004). Based on metabolic needs, heavy metals can often be transformed (e.g., oxidized, reduced, methylated, or complexed) and used as a source of energy, terminal electron acceptors, or enzyme structural elements. Metals can also be taken up from the environment and excreted (in a less toxic form) outside of the cell through various systems of detoxification. Balance and relationships between these processes is mainly determined by the chemistry of the environment (e.g., pH, Eh, inorganic and organic ions, clay and other minerals, humic substances) as well as the abundance of microorganisms engaged in the transformation of heavy metals. For many environmental isolates and pure cultures of bacteria, immobilization processes, such as biosorption (to cell walls, exopolymers, and other structural components), precipitation (as a result of metabolite release; e.g., sulfide), or reduction [e.g., Cr(VI) → Cr(III) or U(VI) → U(IV)] have been described and demonstrated (Gadd, 2010).

This study found that the microbial community structure present in KOW and ZS mats as well as the mechanisms leading to self-purification of the mine waters were significantly different from previously described heavy metal-contaminated environments, such as hot springs, freshwater lake sediments (Aguilera et al., 2010; Gough and Stahl, 2011), or biofilms present either in acid streams or mine drainage (Sánchez-Andrea et al., 2011; Hogsden and Harding, 2012; Streten-Joyce et al., 2013; Mendez-Garcia et al., 2014).

The microbial communities found in KOW and ZS were dominated by filamentous-shaped, sulfur-oxidizing bacteria, such as *Thiothrix, Leptothrix*, and *Beggiatoa* as well as planktonic representatives of *Gammaproteobacteria*, mainly the methane-oxidizing *Methylococcaceae* family, which could be involved in formation of structural backbone of the mats. Those findings are consistent with our previous report on low-throughput biodiversity analysis of Zloty Stok microbial mat community (Drewniak et al., 2012). Together with filamentous bacteria, the microbial mat also contained a large number of cells with other morphotypes (such as cocci and cylindrical forms) that interleaved with the extracellular matrix. Relatively high biodiversity make the mats almost self-sufficient, well-balanced, ecological micro-niches that are similar in some respects to the activated sludge flocks. A similar composition as that in the KOW and ZS communities was previously observed in biofilms that developed in sulfide-rich and metal-poor aquifers in the Frasassi cave system (Italy), which were dominated by *Gammaproteobacteria* with *Beggiatoa*-like and/or *Thiothrix*-like cells (Macalady et al., 2006). The Movile Cave community was also rich in methanotrophic bacteria (Hutchens et al., 2004; Chen et al., 2009) and it was postulated that utilization of C1 products could be an important part of carbon cycling as well as energy production in this environment. Methano- and methylotrophic bacteria are usually found in sediments of temperate and boreal lakes (Borrel et al., 2011; He et al., 2015); however, these bacteria can be also found in highly-contaminated environments. There are several reports concerning the application of methanotrophs in remediation (Oldenhuis et al., 1991; De Marco et al., 2004; Vishnivetskaya et al., 2010; Im and Semrau, 2011), and therefore in this context the role of methanotrophic bacteria in the resistance to heavy metal pollution in the ZS and KOW environments cannot be disregarded.

Post-mining sites like KOW or ZS that have a moderate pH present much greater biodiversity compared to locations with an extreme pH (Baker and Banfield, 2003; Johnson and Hallberg, 2003; Lin et al., 2006). In most acidic mine waters, planktonic prokaryotes are found instead of biofilms. Such communities are generally dominated by the following: (i) chemolithotrophic bacteria of the genera *Acidithiobacillus* and *Leptospirillum*; (ii) heterotrophic genus *Acidiphilium*; and (iii) acidophilic, moderately thermophilic (*Thermoplasmatales*) to extremely thermophilic (*Sulfolobales*) archaea (Baker and Banfield, 2003; Johnson and Hallberg, 2003). Microbial mats present in water contaminated by heavy metals as well as acidic water exhibit completely different phylogeny from those observed in the KOW and ZS mats. For example, metagenomic DNA from mats found in an acidic hot spring in the Yellowstone National Park, USA (Jackson et al., 2001) had dominating sequences that were phylogenetically related to *Hydrogenobacter acidophilus* (requiring elemental sulfur for growth by hydrogen oxidation) and *Desulphurella* sp. (acetate-oxidizing, sulfur-reducing), whereas floating macroscopic filaments of a prokaryotic community found in the extreme acidic environment in Rio Tinto (SW Spain) were dominated by *Acidithiobacillus ferrooxidans, Leptospirillum ferrooxidans, Acidiphilium* spp., and bacteria representing non-acidophilic genera *Aeromonas* and *Acinetobacter* (Garcia-Moyano et al., 2007).

Based on the community structure and phylogenetic comparisons, the following conclusion can be drawn: the phylogeny structure of the microbial communities (mats) found in heavy metal-rich environments is not influenced by these toxic elements, but rather by the pH as well as presence of essential nutrients, such as inorganic electron donors (e.g., sulfur, iron) and the carbon source. Biosorption, precipitation, or reduction are the main mechanisms underlying the properties of microbial mats with regard to water purification from heavy metal ions (Gadd, 2010). The results presented in this study indicate that all of the aforementioned types of immobilization mechanisms occur in the KOW and ZS microbial mats; however, biosorption seems to be the most essential process of metal attenuation in these isolates.

The matrix of the mats was composed of colloidal particles, organic compounds, and metal ions that leached from the rocks and bound to the extracellular polymeric substances (EPS) excreted by the microorganisms. The importance of EPS in biofilm formation and biosorption properties has been described by Cao et al. (2011) and Tourney and Ngwenya (2014); however, the authors mainly refer to single-strain biofilms grown under laboratory conditions. This study analyzed natural occurring biofilms from KOW and ZS communities and confirmed that EPS bonded with clay minerals (i) play crucial roles in biosorption due to the fact that both mats exhibited high retention capacity for heavy metals, which are relatively strong and stably bonded (EDTA was the only eluent that readily contributed to the release of each of the examined elements); (ii) enable capturing and retention of organic and inorganic nutrients; and (iii) significantly affected bacteria cell transport through porous media, as bacteria were found to be uniformly distributed in the structure of the mats. A significant capacity for metal ion binding by microbial mats was proven through the sorption experiments. Notably, ZS presented a much higher sorption capacity for As, Cd, Fe, and Co ions than KOW mats, whereas the latter had a higher capacity for Cu retention. This divergence reflects differences in the chemical properties of EPS excreted by distinct microbial communities.

The hypothesis of biosorption playing a pivotal role in heavy metal removal from mine waters was confirmed through a series of several experiments. First, the EDS analysis showed that metal sorption occurred on the entire surface of the mats, which is consistent with previous studies showing surface distribution of heavy metals as indirect evidence of non-metabolic sorption. Processes such as physical adsorption, ion exchange, and strong binding with carboxylic, sulfate, phosphate, or amino groups were hypothesized to be the main mechanisms of metabolism-independent heavy metal sorption (Kuyucak and Volesky, 1988). The desorption experiment showed that only a strong extractant, such as EDTA, was effective at removing heavy metals from the microbial mats. SEM and EDS analyses also confirmed that the high efficiency of metal sorption by the KOW and ZS mats, similarly to other communities (Lawrence et al., 1998), was attributed to the presence of a highly condensed extracellular matrix, which acts as a physical barrier for metal entrapment and serves as a structure for mineral precipitation. The metagenomic profile of genes responsible for biofilm formation and extracellular polysaccharides production showed that most representatives of the KOW and ZS microbial mats communities were involved in the sorption process, where *Methylococcales* seemed to have the greatest impact, since they were the largest group within both communities. This was confirmed through functional analysis of metagenomic data from both mats, where 2% of the metagenomic reads were annotated to functions involved in biosorption, and genes coding putative HMR proteins only accounted for 1.1% of all reads. On the other hand, the biodiversity analysis showed that both KOW and ZS microbial mats consisted of functional and active dissimilatory sulfate, arsenate, and iron-reducing bacteria representing different genera. This observation corroborated the notion that metabolism-dependent processes, such as precipitation and reduction, are also important in mine water purification. For the ZS and KOW microbial mats, these processes exhibited a lower capacity for heavy metal ion immobilization than biosorption; however, for the microorganisms involved, metabolism-dependent heavy metal removal seemed to be the main driving force for both analyzed communities. Moreover, both microbial mat metagenomes contained representatives of all four main gene groups from heavy metal transformation pathways, and the gene abundance was similar in both analyzed communities (Figure 5).

Based on the comparison of sorption capacity and phylogenetic profiles of the KOW and ZS communities together with previously described microbial mats dominated by *Cyanobacteria* (De Philippis et al., 2011) or *Epsilonproteobacteria* (Naja and Volesky, 2011), it can be explicitly concluded that the morphological structure of a microbial mat is more important than taxonomic distribution. Furthermore, our studies suggested that the biofilm formation and HMR functions are more desirable in microorganisms engaged in self-purification of contaminated waters than the ability to transform heavy metals in respiratory processes. This statement is supported by a comparison of the abundance of genes involved in heavy metal homeostasis and biofilm formation from this study. In both communities, those genes had a similar distribution, with a slight predominance of the functions linked to HMR and electron-donating reactions (Figure 5).

**Figure 9:**
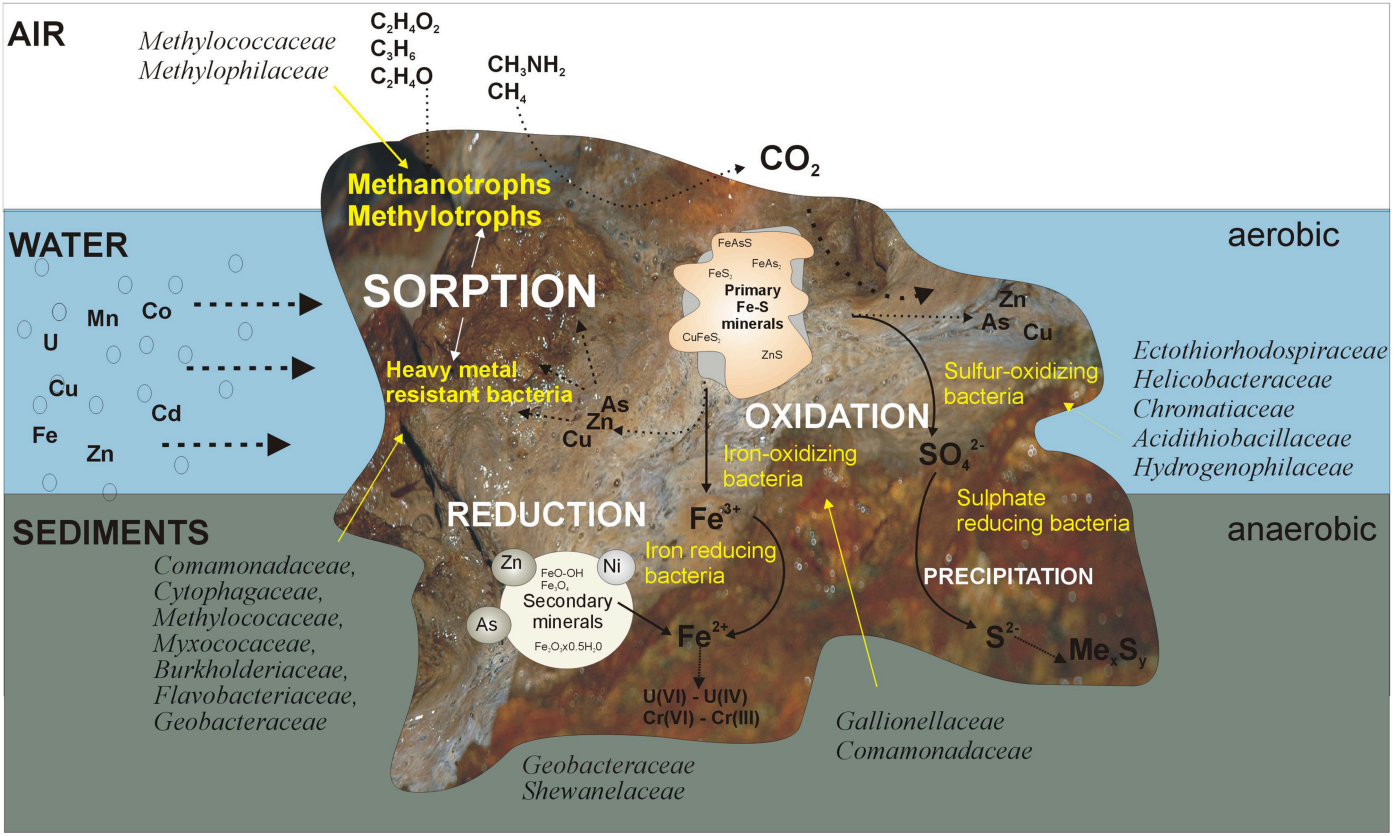
**Proposed mechanisms occurring in the mats that lead to self-purification of the mine waters**. The first barrier trapping heavy metals are EPS bonded with clay minerals. Thus, the producers of an EPS matrix that are able to survive in such an extreme environment are the main driving agent responsible for sorption of heavy metals. Among them, methanotrophs play an important role, since they can utilize C1 substrates and significantly contribute to carbon cycling and energy production in this environment. Immobilization of heavy metals is also enhanced by the activity of microorganisms directly (by oxidation/reduction) and indirectly (by precipitation with sulfides) involved in metal transformations.

This study on the KOW and ZS microbial mats provides insight into the activity of bacteria leading, from biofilm formation to self-purification, of mine waters contaminated with heavy metals. We have attempted to illustrate the structure and function of the mats based on metagenomic and physiological analyses (Figure 9). Although many processes have already been described, further long-term experiments that simulate natural conditions at the laboratory scale or, preferably, *in situ*, are needed in order to gain a deeper understanding of the potential of microbial mats for the natural attenuation of heavy metals. An interesting direction of future research can also be a comparison of KOW and ZS mats with microbial communities inhabiting similar closed environment (eg. caves) but unaltered by human activity.

## Conclusions

This study provide comprehensive insight into biodiversity, biofilm formation and metabolic activity of two microbial mats forming a natural barrier trapping heavy metals leaking from closed mines dewatering systems located in Zloty Stok and Kowary (SW Poland). The presented data and their interpretation lead to the following conclusions:

1. The phylogeny structure of the microbial communities (mats) found in heavy metal-rich environments seems to be not influenced by these toxic elements, but rather by the carbon source and inorganic electron donors (e.g., sulfur, iron).
2. The biofilm formation and HMR functions are more desirable in microorganisms engaged in self-purification of contaminated waters than the ability to transform heavy metals in respiratory processes.
3. Combined functional and metagenomics approaches of CO_2_ assimilation, electron-donating reactions and electron-accepting reactions indicated that microbes involved in the immobilization of heavy metals (metal reduction and co-precipitation), rather than the bacteria engaged in mobilization (eg., chemolithoautotrophs) are the main driving force within the analyzed communities.

## Author contributions

LD, LL conceived and directed the studies. LD, SM participated in microbial mats sampling, chemical analyses, SEM analysis, physiological experiments (sorption, desorption), characterization of anaerobic bacteria and DNA isolation; WB participated in chemical analyses, LL, ASob, and PK designed metagenomics and bioinformatics approach. PK, DA participated in metagenomics DNA isolation, DNA libraries preparation, and sequencing supervision. PK performed all bioinformatics analysis with support from LD, LL, ASob in concluding the data. Funding for chemical and physiological experiments was provided by ASklo, for metagenomics analysis was provided by LL. The manuscript was written by LD, PK and consulted and corrected by LL, ASob, and ASklo. All authors read and approved the final manuscript.

## Funding

This work was supported by strategic research project No. SP/J/3/143045/11 from The National Centre for Research and Development (NCBiR), Poland. The metagenomic part of this work was supported by the EU European Regional Development Fund, the Operational Program Innovative Economy 2007–2013, the project support Agreement POIG.01.01.02-14-054/09-00. Experiments were carried out with the use of CePT infrastructure financed by the European Union—the European Regional Development Fund [Innovative economy 2007–2013, Agreement POIG.02.02.00-14-024/08-00] P.K. is the recipient of a scholarship from the European Social Fund, Human Capital Operational Programme for the execution of the project “Support for bio tech med scientists in technology transfer”; (UDA-POKL.08.02.01-14-041/09), and PRELUDIUM: pre-doctoral grants funded by National Science Centre; 2012/05/N/NZ9/01393.

### Conflict of interest statement

The authors declare that the research was conducted in the absence of any commercial or financial relationships that could be construed as a potential conflict of interest.
