# Supplementary material for: Physiological and Metagenomic Analyses of Microbial Mats Involved in Self-Purification of Mine Waters Contaminated with Heavy Metals
Source: Front Microbiol. 2016 Aug 10;7:1252. doi: 10.3389/fmicb.2016.01252 (PMC4978725; doi:10.3389/fmicb.2016.01252)
Supplement: Supplementary file 6 [file DataSheet1.DOCX]

Supplementary Material

Physiological and metagenomic analyses of microbial mats involved in self-purification of mine waters contaminated with heavy metals

Lukasz Drewniak^1*^#, Pawel S Krawczyk^2^#, Sebastian Mielnicki^1^, Dorota Adamska^2^, Adam Sobczak^2^, Leszek Lipinski^2^, Weronika Burec-Drewniak^3^, Aleksandra Sklodowska^1^

^1^ Laboratory of Environmental Pollution Analysis, Faculty of Biology, University of Warsaw, Miecznikowa 1, 02-096 Warsaw, Poland; (*correspondence: ldrewniak@biol.uw.edu.pl)

^2^ Laboratory of RNA Biology and Functional Genomics, Institute of Biochemistry and Biophysics, PAS, Pawinskiego 5a, 02-106 Warsaw, Poland;

^3^ Polish Geological Institute-National Research Institute, Rakowiecka 4, 00-975 Warsaw, Poland

**# Equal contributions**

***Correspondence:**

Dr. Lukasz Drewniak

University of Warsaw, Faculty of Biology,

Laboratory of Environmental Pollution Analysis,

Miecznikowa 1,

Warsaw, 02-096, Poland

[ldrewniak@biol.uw.edu.pl](mailto:ldrewniak@biol.uw.edu.pl)

# Supplementary Figures and Tables

## Supplementary Figures

**
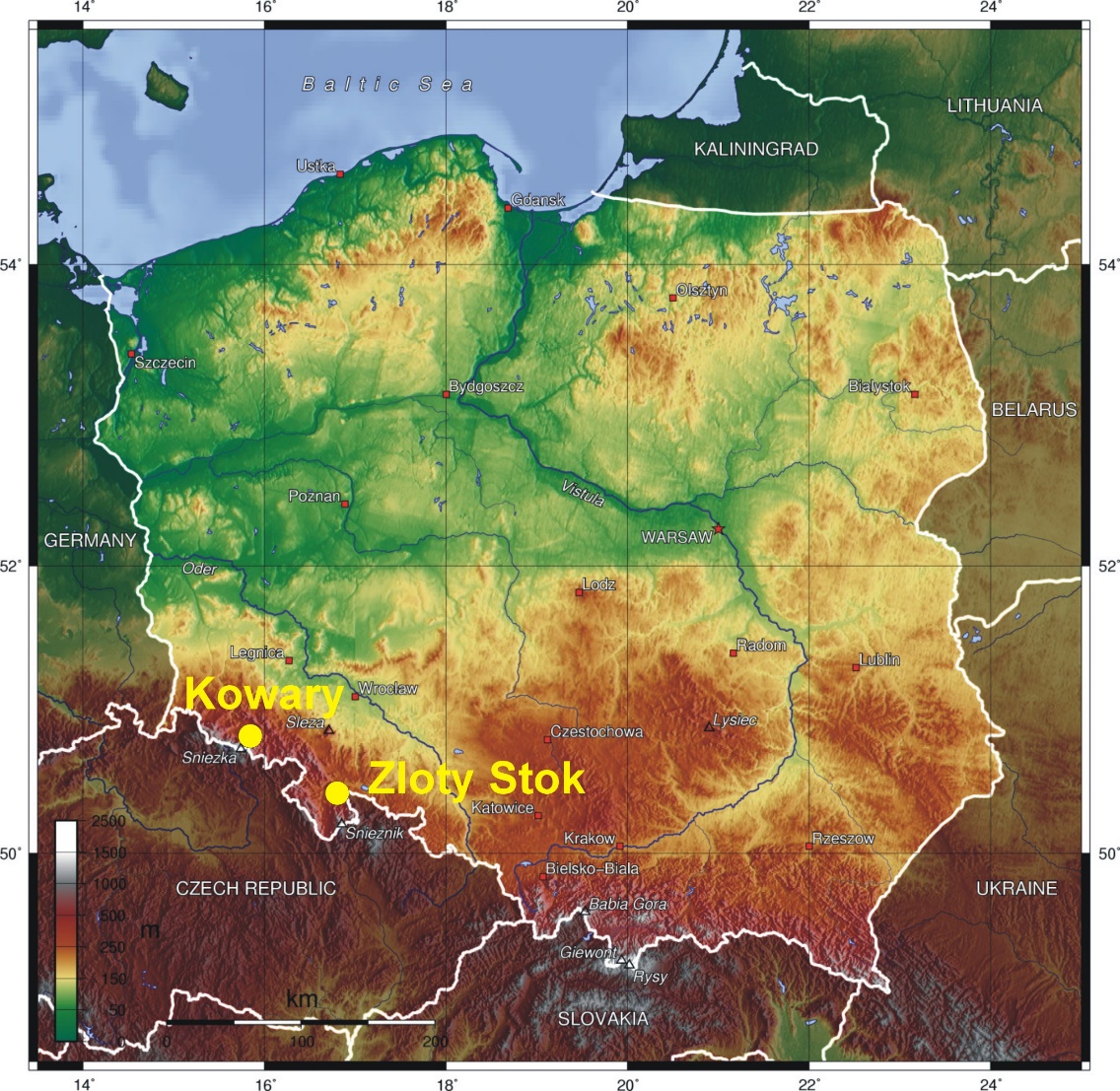
**

**Supplementary Figure S1. Map presenting location of gold mine in Zloty Stok and uranium mine in Kowary.**

**
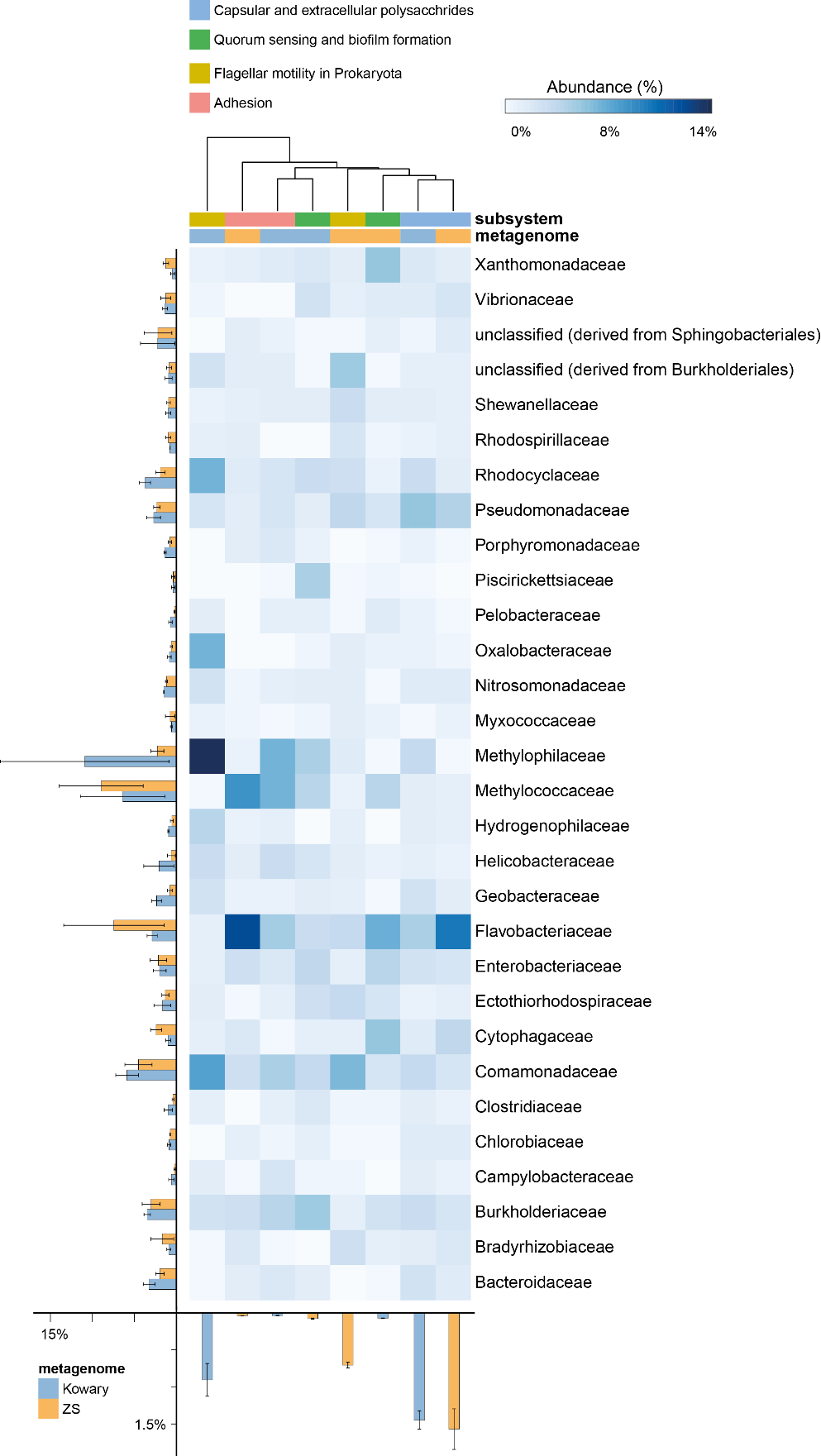
**

**Supplementary Figure S2. Taxonomic distribution of reads assigned to capsular and extracellular polysaccharides subsystem.** Heatmap shows relative abundance of reads classified to given family in particular subsystem. On the left and in the bottom total abundances in metagenomes are shown for families and subsystems, respectively. Subsystems where clustered using average neighbor (UPGMA) method**.**

**
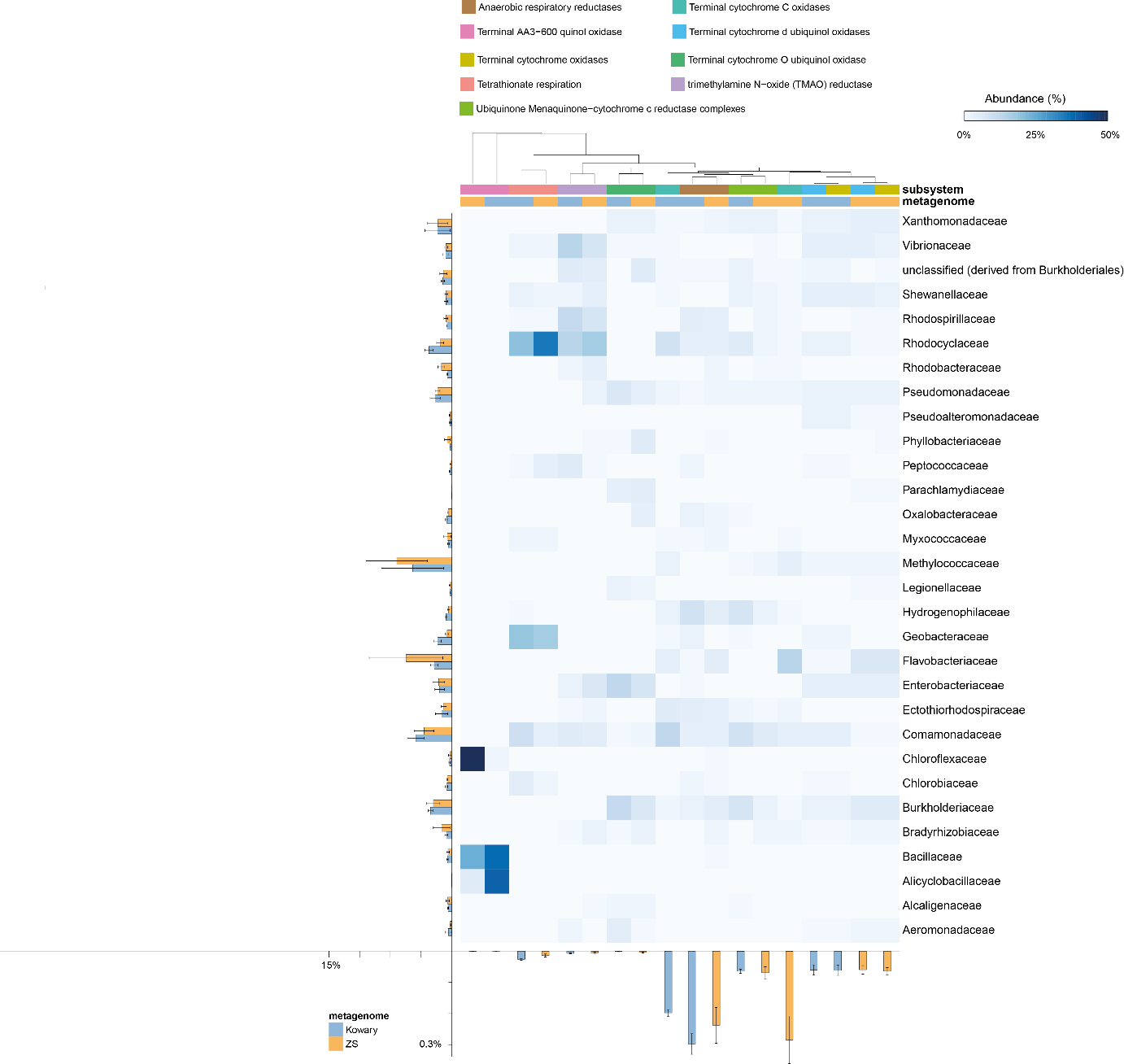
**

**Supplementary Figure S3.** **Taxonomic distribution of reads assigned to electron accepting reactions subsystems.** Heatmap shows relative abundance of reads classified to given family in particular subsystem. On the left and in the bottom total abundances in metagenomes are shown for families and subsystems, respectively. Subsystems where clustered using average neighbor (UPGMA) method.

**
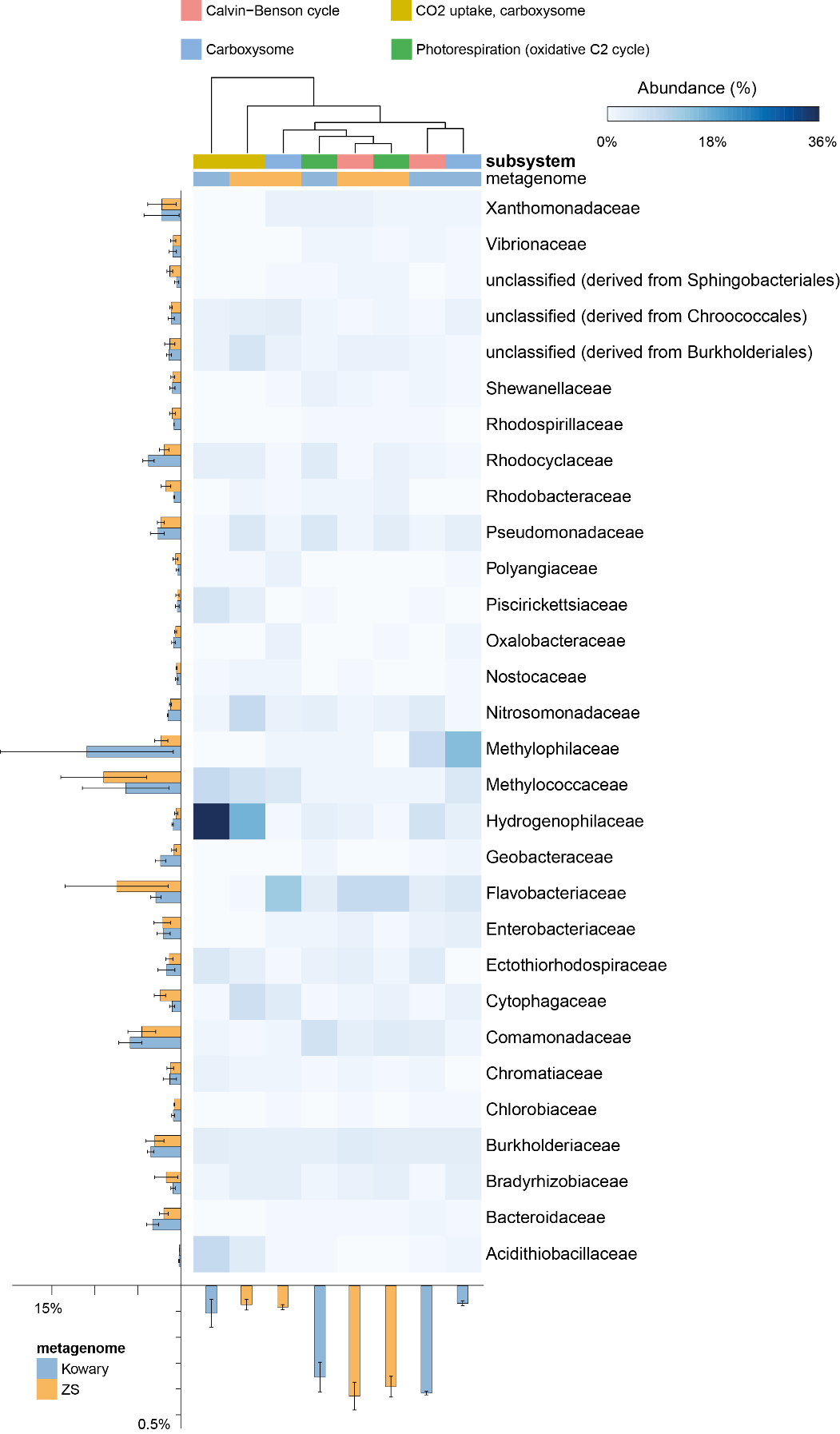
**

**Supplementary Figure S4.** **Taxonomic distribution of reads assigned to CO_2_ fixation subsystems.** Heatmap shows relative abundance of reads classified to given family in particular subsystem. On the left and in the bottom total abundances in metagenomes are shown for families and subsystems, respectively. Subsystems where clustered using average neighbor (UPGMA) method.

**
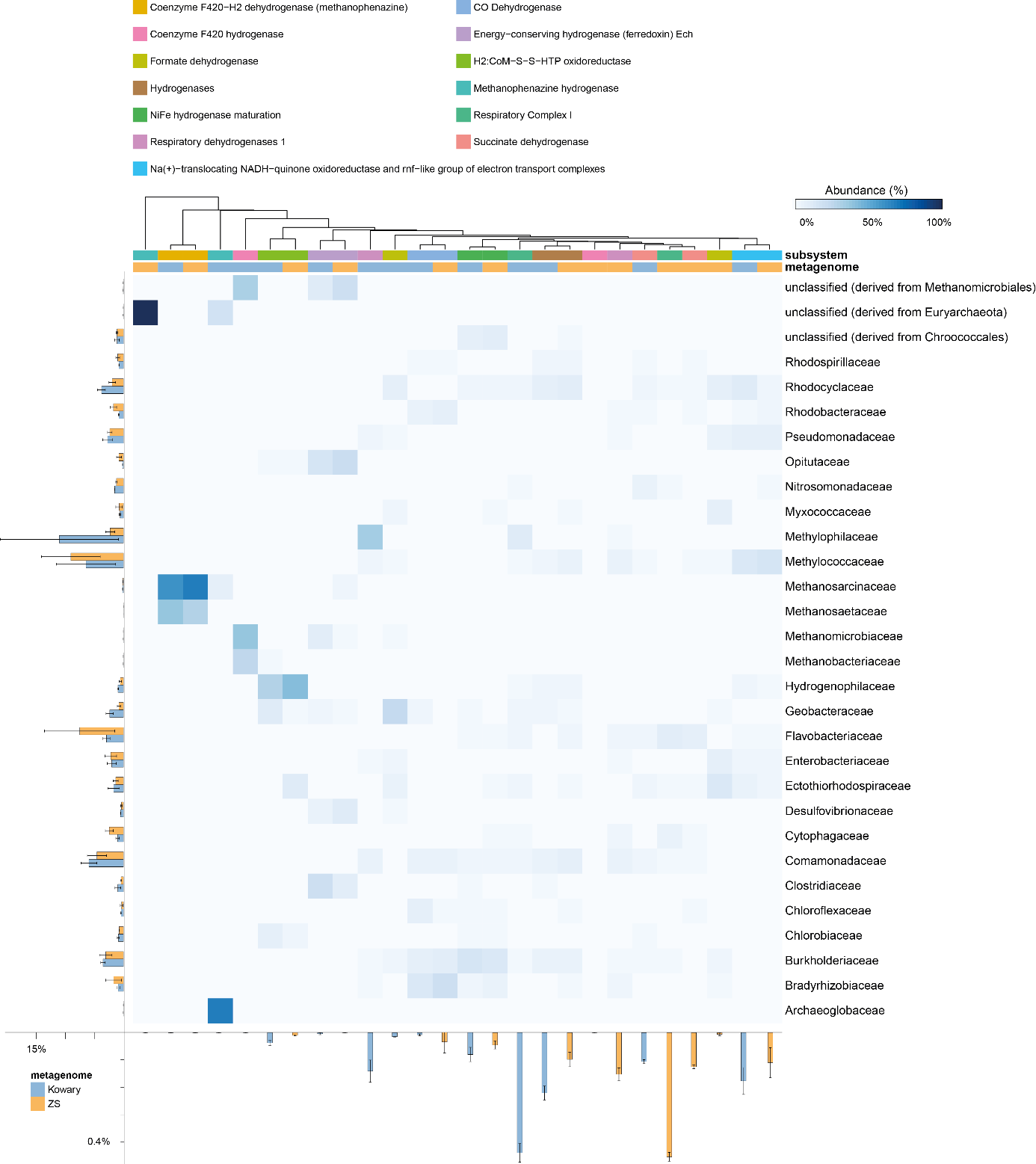
**

**Supplementary Figure S5.** **Taxonomic distribution of reads assigned to electron donating reactions subsystems.** Heatmap shows relative abundance of reads classified to given family in particular subsystem. On the left and in the bottom total abundances in metagenomes are shown for families and subsystems, respectively. Subsystems where clustered using average neighbor (UPGMA) method.

**
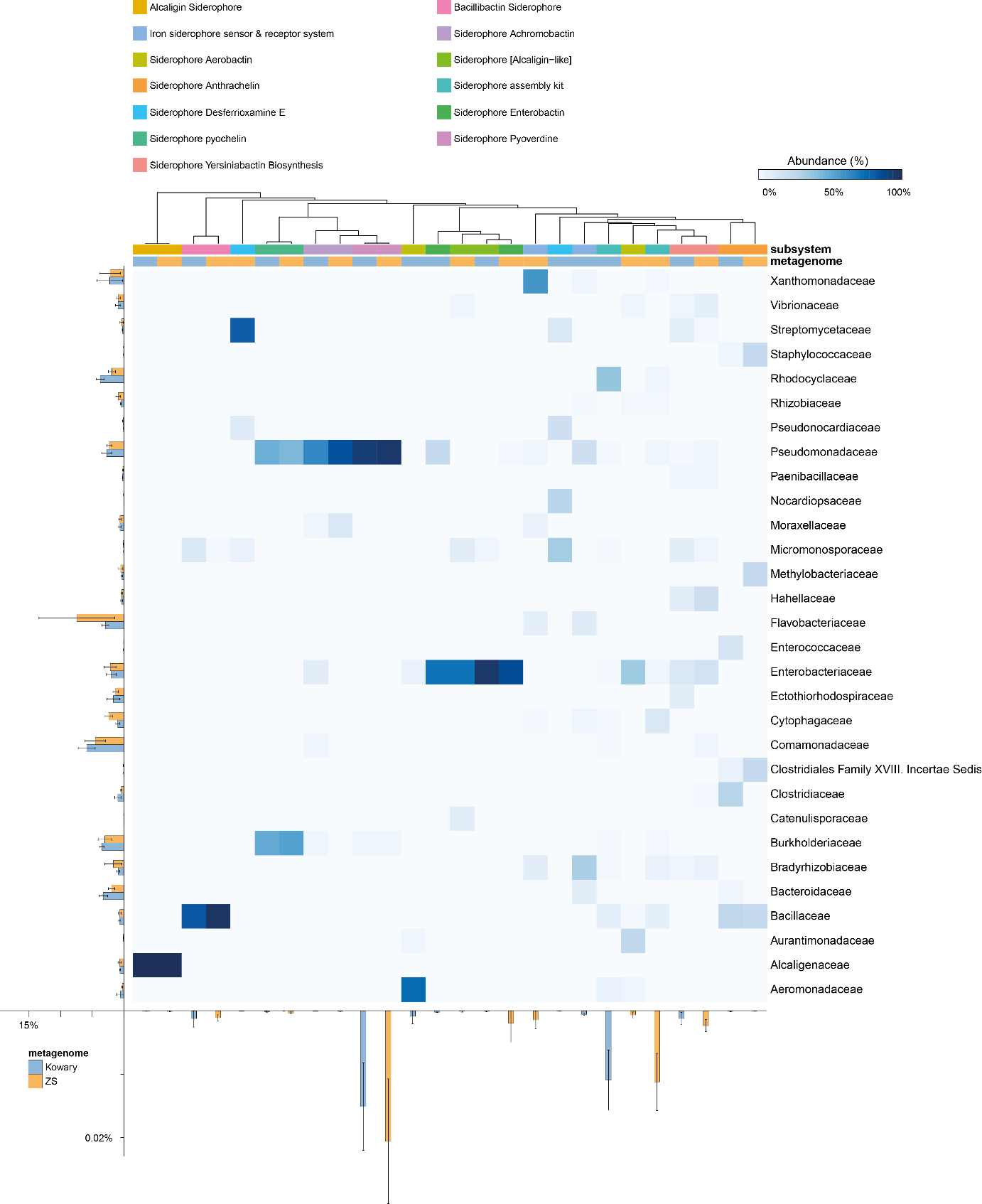
**

**Supplementary Figure S6.** **Taxonomic distribution of reads assigned to siderophores subsystems.** Heatmap shows relative abundance of reads classified to given family in particular subsystem. On the left and in the bottom total abundances in metagenomes are shown for families and subsystems, respectively. Subsystems where clustered using average neighbor (UPGMA) method.

## Supplementary tables

**Supplementary Table S1. Sequencing statistics.** Sheet Enrichment amplicon sequencing stats contains sequencing statistics of enrichment amplicon data. Collapsed reads is a number of unique MT categories. In Enrichment amplicon taxonomic assignments RDP taxonomic assignments of obtained OTUs, as well as their counts in analyzed enrichment cultures are shown. Shotgun sequencing stats include shotgun statistics obtained from the MG-RAST

**Supplementary Table S2. Genbank-based biodiversity analysis table.** Genbank based best-hit classification of analyzed metagenomes calculated using MG-RAST were loaded to STAMP and compared using Fisher's exact test, Newcombe-Wilson confidence interval method and Benjamini-Hochberg FDR correction.

**Supplementary Table S3. KEGG annotation table.** KEGG based MG-RAST profiles of analyzed metagenomes were loaded to STAMP and compared using Fisher's exact test, Newcombe-Wilson confidence interval method and Benjamini-Hochberg FDR correction.

**Supplementary Table S4. Subsystem annotation table.** Subsystem based MG-RAST profiles of analyzed metagenomes were loaded to STAMP and compared using Fisher's exact test, Newcombe-Wilson confidence interval method and Benjamini-Hochberg FDR correction.

**Supplementary Table S5. Taxonomic distribution of cytochromes c.** Metagenomic reads classified in MG-RAST as cytochromes c were found using keyword search in IMG annotation table. Obtained reads were re-classified taxonomically using Workbench function and Genbank reference database. Obtained profiles were loaded to STAMP and compared using Fisher's exact test, Newcombe-Wilson confidence interval method and Benjamini-Hochberg FDR correction.
